# Supplementary material for: Bi-allelic loss-of-function variants in BCAS3 cause a syndromic neurodevelopmental disorder
Source: Am J Hum Genet. 2021 May 21;108(6):1069–82. doi: 10.1016/j.ajhg.2021.04.024 (PMC8206390; doi:10.1016/j.ajhg.2021.04.024)
Supplement: Document S1. Figures S1–S4, Tables S1 and S2, and supplemental methods [file mmc1.pdf]

The American Journal of Human Genetics, Volume *108*

Supplemental information

Bi-allelic loss-of-function variants in *BCAS3* cause a syndromic neurodevelopmental disorder

Holger Hengel, Shabab B. Hannan, Sarah Dyack, Sara B. MacKay, Ulrich Schatz, Martin Fleger, Andreas Kurringer, Ghassan Balousha, Zaid Ghanim, Fowzan S. Alkuraya, Hamad Alzaidan, Hessa S. Alsaif, Tadahiro Mitani, Sevcan Bozdogan, Davut Pehlivan, James R. Lupski, Joseph J. Gleeson, Mohammadreza Dehghani, Mohammad Y.V. Mehrjardi, Elliott H. Sherr, Kendall C. Parks, Emanuela Argilli, Amber Begtrup, Hamid Galehdari, Osama Balousha, Gholamreza Shariati, Neda Mazaheri, Reza A. Malamiri, Alistair T. Pagnamenta, Helen Kingston, Siddharth Banka, Adam Jackson, Mathew Osmond, Care4Rare Canada Consortium, Genomics England Research Consortium, Angelika Rieß, Tobias B. Haack, Thomas Nägele, Stefanie Schuster, Stefan Hauser, Jakob Admard, Nicolas Casadei, Ana Velic, Boris Macek, Stephan Ossowski, Henry Houlden, Reza Maroofian, and Ludger Schöls

Figure S1

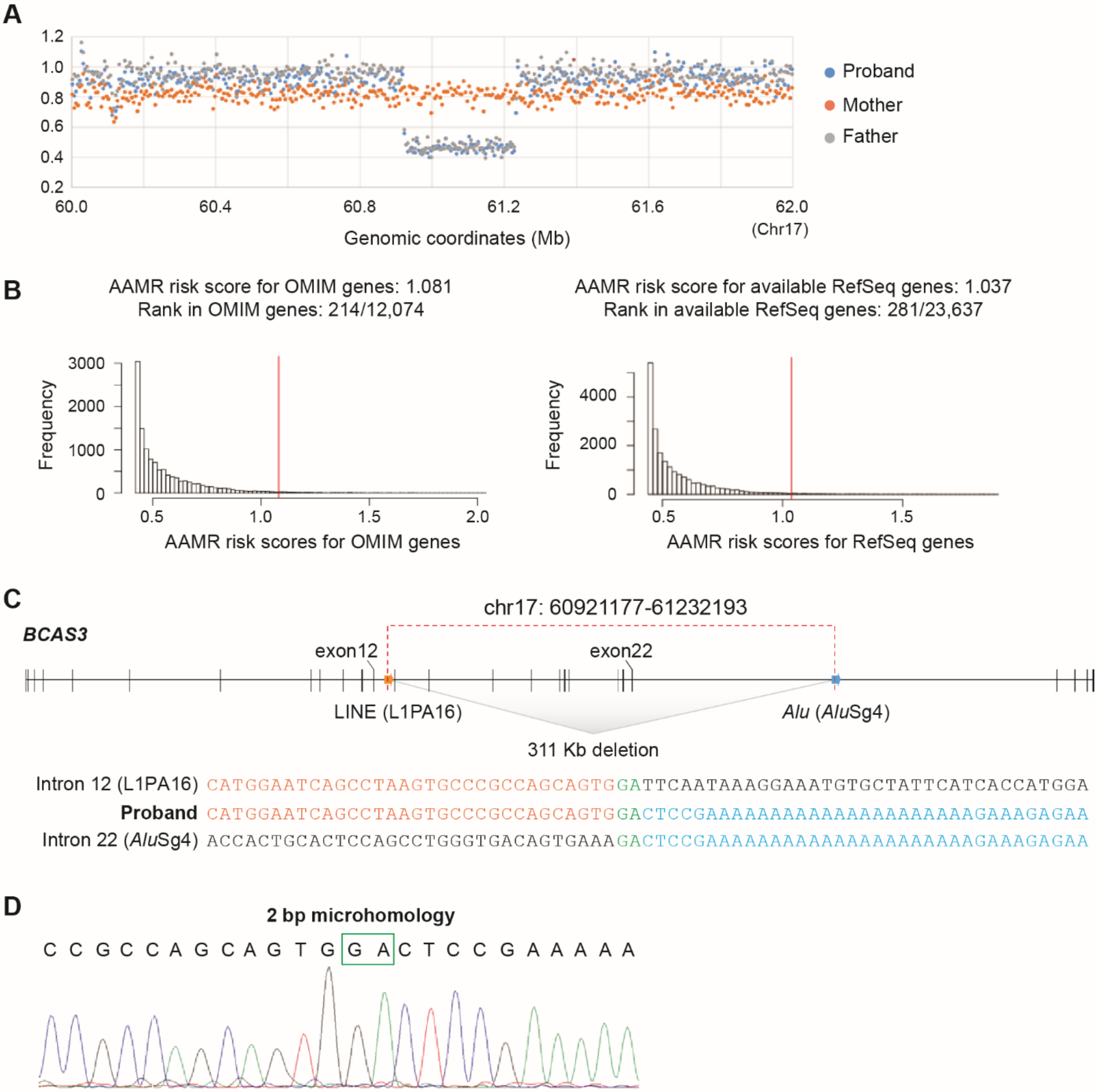

A
1.2
1.0
0.8
0.6
0.4
0.2
60.0
60.4
60.8
61.2
61.6
62.0
(Chr17)
Genomic coordinates (Mb)
Proband
Mother
Father
B
AAMR risk score for OMIM genes: 1.081
Rank in OMIM genes: 214/12,074
AAMR risk score for available RefSeq genes: 1.037
Rank in available RefSeq genes: 281/23,637
Frequency
3000
2000
1000
0
0.5
1.0
1.5
2.0
AAMR risk scores for OMIM genes
Frequency
4000
2000
0
0.5
1.0
1.5
AAMR risk scores for RefSeq genes
C
chr17: 60921177-61232193
BCAS3
exon12
exon22
LINE (L1PA16)
Alu (AluSg4)
311 Kb deletion
Intron 12 (L1PA16) CATGGAATCAGCCTAAGTGCCCGCCAGCAGTGGATTCAATAAAGGAAATGTGCTATTCATCACCATGGA
Proband CATGGAATCAGCCTAAGTGCCCGCCAGCAGTGGACTCCGAAAAAAAAAAAAAAAAAAAAAGAAAGAGAA
Intron 22 (AluSg4) ACCACTGCACTCCAGCCTGGGTGACAGTGAAAGACTCCGAAAAAAAAAAAAAAAAAAAAAGAAAGAGAA
D
2 bp microhomology
C C G C C A G C A G T G G A C T C C G A A A A A

Figure S1: A 311 Kb exonic deletion CNV allele generated by *Alu* – LINE rearrangement. **a** The 311 kb deletion identified by whole genome sequencing in F8. The read counts for non-overlapping 5 kb windows across the region was normalized to a control subject and shows evidence for copy number loss across ~ 300 Kb genomic interval. **b** The AluAluCNVPredictor tool (http://alualucnvpredictor.research.bcm.edu:3838/)[1] revealed the *Alu-Alu* mediated rearrangement (AAMR) score, a potential measure of susceptibility to genomic instability based on repetitive sequence gene architecture for that given gene, for *BCAS3* to be 1.081 for OMIM genes and 1.037 for RefSeq genes; a score > 0.6 implicates susceptibility to AAMR. **c** Schematic representation of exonic structure of *BCAS3*. Breakpoint sequence analysis for *BCAS3* deletions using the MultAlin alignment tool[2] is also shown. Proximal reference sequence and patient breakpoint sequences that match the proximal reference sequence are shown in orange, the distal reference sequence and patient breakpoint sequences that match the distal reference sequence are shown in blue. Note, 2 bp microhomology (GA) at the junction (shown in green). The 311 Kb deletion involving 10 exons identified in F8-II.1 presumably results from a Microhomology-Mediated Break-Induced Replication (MMBIR) event[3] generating the *BCAS3* intragenic deletion. **d** Sanger confirmation of the break point junction in F8-II.1.

Figure S2

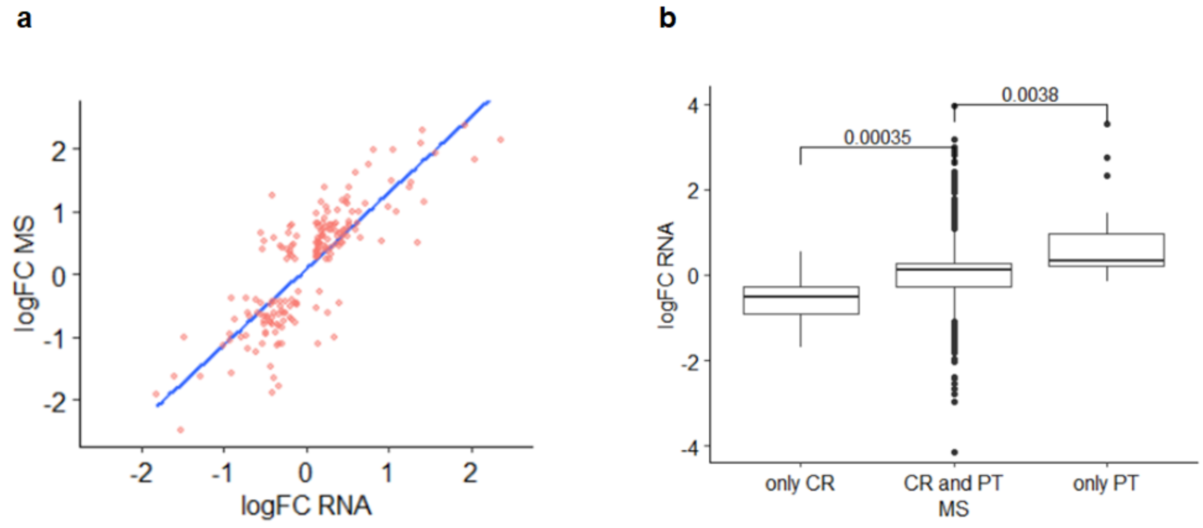

Figure S2: Correlation of RNA-Seq and NanoLC-MS/MS data

a Correlation of significant up/or downregulated genes at the mRNA level with significant up-/or downregulated proteins in MS. **b** Bar chart of fold changes in RNA-Seq data from proteins that were measured using nanoLC-MS/MS in only controls, in controls and patients and in only patients.

Figure S3

a

Driver	Expression(25°C) *rudhira* -RNAi-a	Lethality	Adult phenotype
control	n/a	-	Healthy
daughterless	Ubiquitous	Embryonic	-
elav	Pan-neuronal	Embryonic	-
Appl	Pan-neuronal	-	Weak, short-lived
GMR	Eye	-	Depigmentation
D42	Motor neuronal	Larval	-
ppk	Sensory neuronal	-	Healthy
repo	Pan-glial	Larval	-
Mhc	Muscles	Larval/Pupal	-

b

Driver	Expression (29°C) *rudhira-RNAi-a*	Lethality	Adult phenotype
control	n/a	-	Healthy
daughterless	Ubiquitous	Embryonic	-
elav	Pan-neuronal	Embryonic	-
Appl	Pan-neuronal	-	Paralyzed
GMR	Eye	Pupal	-
D42	Motor neuronal	Larval	-
ppk	Sensory neuronal	-	Healthy
repo	Pan-glial	Larval	-
Mhc	Muscles	Larval	-

c

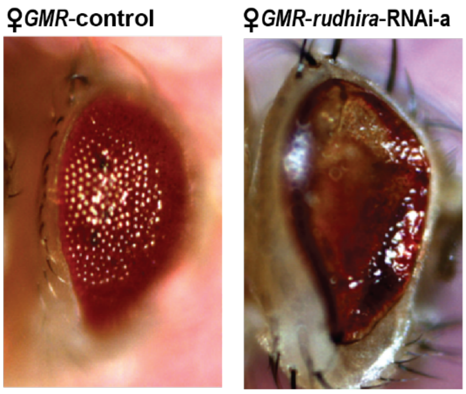

d

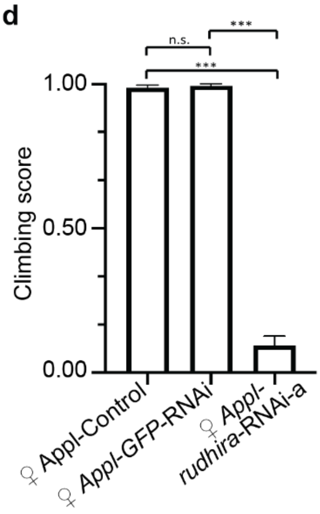

e

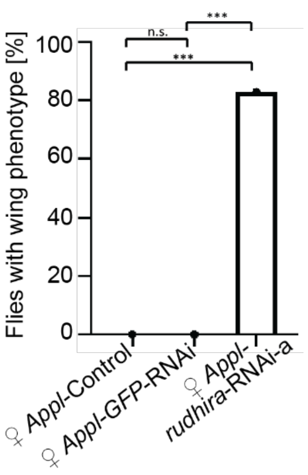

f

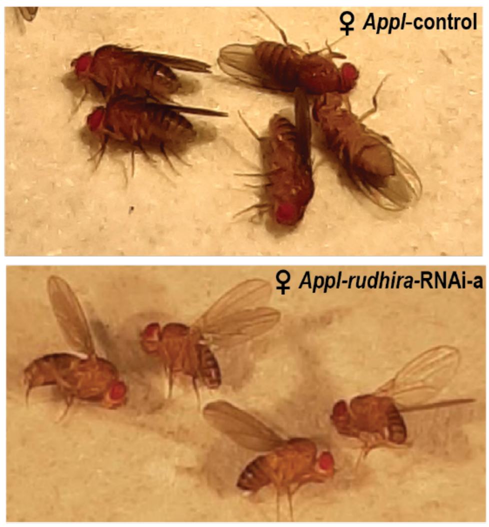

g

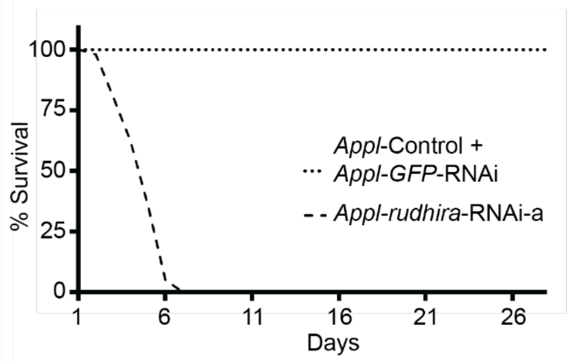

Figure S3: *Drosophila* loss-of-function model *based on the ruhdira-RNAi-a line.*

a Using the UAS-*rudhira*-RNAi-a line*,* knockdown of *rudhira* using different tissue specific neuronal and nonneuronal Gal4 driver lines at 25°C. Ubiquitous (*daughterless*), pan-neuronal (elav), motor neuroal (*D42*-Gal4), glial (*repo*-Gal4) and muscle (*Mhc*-Gal4) specific drivers caused lethality, whereas no effect was observed using a sensory neuron driver (*ppk*-Gal4). Adult flies with specific phenotypes were observed under the *Appl*-Gal4 (pan neuronal, active beginning in the larval stage) and *GMR*-Gal4 (eye-specific) drivers. **b** A higher temperature at 29°C shifted lethality to an earlier developmental stage. **c** Representative images of eyes when the Canton S line or *rudhira*-RNAi-a was crossed with the *GMR*-Gal4 driver at 25°C. The rough eye phenotype that was observed in *GMR-rudhira*-RNAi-a flies has been widely used to study the effect of gene knockdown or overexpression on neuronal dysfunction and neurodegeneration in *Drosophila*[4]. **d** Using a negative geotaxis assay, adult *Appl-rudhira*-RNAi-a and *Appl*-control flies were assayed for their ability to climb. *Appl-rudhira*-RNAi-a flies had a severe locomotion defect with impaired climbing abilities (Video S1-S3). Crossings were set up at 25°C. More than 50 flies were analyzed per genotype. **e** Impaired wing posture with wings perpendicular to the body in *Appl-ruhdira*-RNAi-a flies, while wings remained normal in *Appl*-control and *Appl*-GFP-RNAi flies. 120 *Appl*-control flies, 101 *Appl*-GFP-RNAi, and 128 *Appl-ruhdira*-RNAi-a were analyzed. **f** Representative images of the wing phenotype of *Appl-rudhira*-RNAi-a flies compared to *Appl*-control flies. **g** Compared to *Appl*-control and *Appl-GFP*-RNAi flies*, Appl-rudhira*-RNAi-a flies obtained at 25°C showed a reduced life span with a maximum life expectancy of 7 days. 100 flies were followed up in all three groups. While all *Appl-rudhira*-RNAI-a flies were dead after 7 days, all *Appl*-control or *Appl-GFP*-RNAi flies were still alive after 26 days of follow-up (log-rank $p<0.001$).

Figure S4

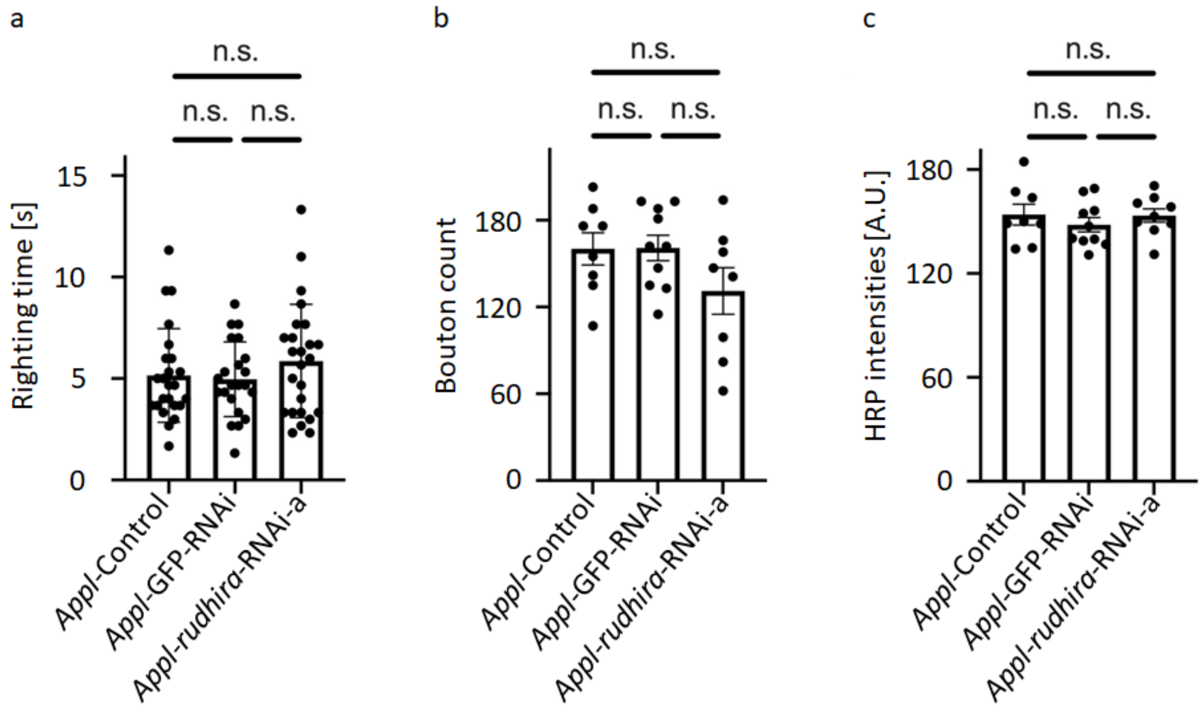

Figure S4: Characterization of control, GFP-RNAi, and *rudhira*-RNAi-a knockdown larvae using the *Appl*-Gal4 driver. a Comparison of the righting times between the *Appl*-control strain, *Appl*-GFP-RNAi and *Appl-rudhira*-RNAi-a larvae. The *Appl*-control group represents F1 female larvae following the cross between *Appl*-Gal4 with background control strain (BDSC #36304). *Appl-GFP*-RNAi group represents F1 female larvae following the cross between *Appl*-Gal4 with *GFP*-RNAi (BDSC #9331). More than 20 individual larvae were analyzed per genotype. No significant differences were observed between any groups with Kruskal-Wallis (p=0.62) followed by Dunn's multiple comparisons test, while there was a trend towards a slower righting time in the *Appl-rudhira*-RNAi-a larvae. **b** Bouton counts from *Appl*-control, *Appl-GFP*-RNAi and *Appl-rudhira*-RNAi-a larvae. N between 8-10 NMJs, n represents NMJs from distinct larvae. No significant differences were observed between any groups with Kruskal-Wallis (p=0.37) followed by Dunn's multiple comparisons test, while there was a trend towards less boutons in the *Appl-rudhira*-RNAi-a larvae. **c** HRP intensities quantified from neuromuscular junctions of *Appl*-control, *Appl-GFP*-RNAi and *Appl-rudhira*-RNAi-a larvae. N between 8-10 NMJs, n represents NMJs, each from a distinct larvae. No significant differences were observed between any groups with Kruskal-Wallis (p=0.61) followed by Dunn's multiple comparisons test **.**

Table S1: Genetic characteristics of identified BCAS3 variants

Family	Chr	Start	End	Ref	Alt	Variant (NM_001099432.3)	gnomAD (v2.1.1)	SIFT	Polyphen2 (HumVar)	CADD (phred)	GERP++	phyloP (100way)	ClinVar Accession
F1	17	60679530	60679530	C	T	c.73C>T, p.Gln25*	.	0	.	38	4.53	5.688	SCV001470694
F2	17	60889759	60889759	T	G	c.726T>G, p.Tyr242*	.	1	.	38	5.99	1.95	SCV001470695
F3	17	60990206	60990206	C	G	c.1457C>G, p.Ser486*	.	0.08	.	39	5.38	6.995	SCV001470696
F4	17	61034683	61034683	C	T	c.1700C>T, p.Pro567Leu	2.03E-05	0.3	0.997	26.4	6.06	7.487	SCV001470697
F4	17	61034712	61034712	G	A	c.1729G>A, p.Gly577Arg	1.61E-05	0.14	0.999	24.1	6.06	9.476	SCV001470698
F5	17	61078384	61078384	C	T	c.2227C>T, p.Gln743*	.	0.14	.	40	5.61	7.598	SCV001470699
F6	17	60947264	60947264	T	-	c.1133delT, p.Val378fs*5	.	.	.	.	.	.	SCV001470700
F7	17	60868629	60868629	T	-	c.530delT, p.Met177fs*23	.	.	.	.	.	.	SCV001470701
F8	17	60921177	61232193	311 kb deletion		c.994-3230_2426-136134del, p.?	.	.	.	.	.	.	SCV001470702
F8	17	61040896	61040899	AGTA	-	c.2074+4_2074+7delAGTA, p.?	.	.	.	.	.	.	SCV001470703
F9	17	60747213	60747213	C	T	c.337C>T, p.Gln113*	.	0.29	.	37	5.36	5.456	SCV001548239
F9	17	60868675	60868675	C	A	c.576C>A, p.Cys192*	.	1	.	37	2.59	2.724	SCV001548240
Candidate variant from Hu et al.	17	61084564	61084564	G	C	c.2470G>C, p.Gly824Arg	.	0.01	0.804	18.37	5.92	9.187	n/a

Table S2: Analysis and pathological findings in available MRI studies

Proband	F1-II.1	F1-II.2	F2-II.3	F2-II.2	F2-II.7	F3-II.1	F4-II.1	F5-II.1	F5-II.2	F6-II.2	F6-II.1	F6-II.1	F7-II.1	F7-II.2	F9-II.1
Age	9 yrs.	5 yrs.	21 yrs.	22 yrs.	7 yrs.	14 mo.	5 mo.	9 yrs.	4/1/2 yrs.	4 yrs.	1 yr.	7 yrs.	7 yrs.	6 yrs.	11 yrs.
Medulla oblongata	0	0	0	0	0	0	1	0	0	0	0	0	0	0	0
Pons	0	0	0	0	0	0	2	0	0	0	0	0	0	0	0
Mesencephalon	0	0	0	0	0	0	0	0	0	0	0	0	0	0	0
Cerebellar vermis	0	1	1	1	1	0	0	1	1	2	1	1	1	1	1
Cerbellar hemispheres	0	0	1	1	0	0	1	1	1	0	0	0	0	0	0
Corpus callosum genu	0	0	0	0	0	0	2	0	1	1	1	1	0	0	2
Corpus callosum body	1	1	1	1	1	0	2	0	2	1	1	1	1	0	2
Corpus callosum splenium	2	2	2	2	1	2	2	2	2	2	2	2	1	1	2
Frontal cortex	0	0	1	2	1	1	1	0	1	2	1	2	1	0	0
Parietal cortex	0	0	2	2	1	1	1	0	1	2	1	1	1	0	0
Occipital cortex	0	0	2	1	1	0	1	0	1	2	0	1	1	0	0
Temporal cortex	0	0	1	0	0	0	1	0	1	2	0	2	1	0	0
Myelination frontal	1	1	0	0	1	1	2	1	1	2	1	0	1	1	1
Myelination parietal	2	2	0	0	1	1	2	1	1	2	1	0	1	1	1
Myelination occipital	1	1	0	0	0	0	2	1	1	2	1	0	1	0	1
Myelination temporal	1	1	0	0	0	1	2	1	1	1	1	0	1	0	1
Basal ganglia	0	0	0	0	0	0	1	0	0	0	0	0	0	0	0
Angiography	n/a	n/a	0	0	0	n/a	n/a	n/a	Vein of Galen aneurysmal malformation	n/a	n/a	n/a	n/a	n/a	n/a

Evaluation of the pathological findings (atrophy or myelination deficits): 0 = normal, 1 = slightly pathological, 2 = clearly pathological.

Supplemental Methods

Drosophila husbandry and strains

The following additional Gal4 driver lines were used: *ppk*-Gal4 (BDSC #32078, #32079), *Mhc*-Gal4 (BDSC #55133), *GMR*-Gal4 (from Aaron Voigt, Department of Neurology in RWTH Aachen University) and *repo*-Gal4 (from Christian Klämbt, University of Münster).

Immunohistochemistry

Immunohistochemistry at the neuromuscular junction in aged-matched L3 larvae was performed as previously described [5, 6]. Only female larvae were used for dissection. In brief, larvae were removed from food with a brush and washed gently with tap water at room temperature. Larvae were then immobilized on a dissection pad with dissection pins and Ca2+ free HL-3 solution (on ice) were added to cover the larvae. Dissection scissors were used for dissection to expose body wall muscles by cutting along the dorsal side midline of the larvae. Next, inner organs were gently removed, HL-3 was removed and 4% paraformaldehyde in phosphate-buffered saline (PBS) was added for 10min for fixation. For blocking solution, PBS supplemented with 0.05% Triton X100 (PBT) was utilized, and 5% normalized goat serum in PBT was used (1 hour at room temperature). Goat antihorseradish peroxidase (HRP) conjugated with Cy3 (Dianova) was added and incubation performed for 2h at room temperature. HRP antibody incubation was performed was performed in 5% normalized goat serum in PBT. Samples were washed in PBS (3 times, 20 mins) prior to mounting in Vectashield (Vector Laboratories).

Microscopy and image analysis

Microscopy and image analysis were performed as previously described [7]. Fixed samples were imaged on a Zeiss LSM 710 confocal microscope equipped with 405, 440, 488, 514, 561 and 633 Laser Lines and ConfoCor 3 Scanhead. Imaging was performed using a 40X oil objective with 1.3 N. A.; Voxel Size: 100nm, 100nm, 500 nm; pinhole: 1 AU, average: 2. Samples were imaged while avoiding oversaturation of signals. For quantitative comparisons of intensities,

common settings were chosen for all parameters, including the speed of scanning, frame size, laser intensities, and digital gain. NMJ from muscle 6/7 in segment 3 from either the left or right hemisegment were imaged. Image processing at the larval neuromuscular junction was performed using the open source image processing software Fiji (https://imagej.net/Fiji, version 1.0). Boutons were counted manually using the HRP signal that stains neuronal membranes in nerve terminals. A binary 0-255 mask was generated using the HRP signal to quantify intensities. The same settings were used across all genotypes for generation of the HRP masks. Binary masks were used to do a minimum image calculation with HRP raw data that were background subtracted (the same background was subtracted across all genotypes). Intensities were analyzed from larvae that were dissected on the same day, incubated together in the same concentration of antibodies, and imaged using identical settings on the same imaging session at the confocal microscope. 8-10 NMJs from muscle 6/7 in segment 3, each from a distinct larvae, were analyzed per genotype.

Adult eye phenotypes

Three- to five-day-old adult flies from crosses between GMR-Gal4 with stock 51691 or Canton S as a control were frozen for 1 h at −20°C and then mounted on microscope slides. Eye pictures were taken using a Zeiss AxioImager Z1 microscope and AxioVision software.

Climbing assay

The climbing assay was performed as previously described[6, 8]. Flies were collected from all genotypes within 0-48h after eclosion. Flies were monitored by analyzing their ability to climb 6 cm in 14 s. . A successful attempt was scored as 1, and failure to reach the top as 0. Each fly was assessed three times to calculate the average climbing score (1 if all attempts three are successful, 0.67 if two out of three attempts are successful, and 0.33 if one out of three attempts are successful). Per genotype 50 female flies were analyzed.

Righting assay

Larval righting assay was performed as previously described[8, 9]. Only female L3 larvae from each genotype were used for the righting assay. In brief, larvae were removed from food and gently washed with tap water at room temperature. Before proceeding with the experiments, larvae were placed for 10min on an agar plate to acclimatize to experimental conditions. For the assay, larvae were turned one at a time and put upside down on agar plates. The time required to regain normal posture (righting) and initiate the first contraction wave was recorded using a stopwatch. Each larva was assayed three times, and the average righting time per trial was used for analysis. At least 20 larvae were analyzed per genotype.

Longevity assay

Emerged flies were collected within 24 h after eclosion and were kept at 25°C in a 12 h day/night cycle. Flies were transferred to vials containing fresh food media every day without anaesthetization. 100 flies were followed per genotype.

Supplemental References

1. Song X, Beck CR, Du R, et al. Predicting human genes susceptible to genomic instability associated with Alu/Alu-mediated rearrangements. Genome Res 2018;28(8):1228-1242.
2. Corpet F. Multiple sequence alignment with hierarchical clustering. Nucleic Acids Res 1988;16(22):10881-10890.
3. Mayle R, Campbell IM, Beck CR, et al. DNA REPAIR. Mus81 and converging forks limit the mutagenicity of replication fork breakage. Science 2015;349(6249):742-747.
4. Lenz S, Karsten P, Schulz JB, Voigt A. Drosophila as a screening tool to study human neurodegenerative diseases. J Neurochem 2013;127(4):453-460.
5. Zhang YV, Hannan SB, Kern JV, et al. The KIF1A homolog Unc-104 is important for spontaneous release, postsynaptic density maturation and perisynaptic scaffold organization. Sci Rep 2017;7:38172.
6. Zhu JY, Hannan SB, Drager NM, et al. Autophagy inhibition rescues structural and functional defects caused by the loss of mitochondrial chaperone Hsc70-5 in Drosophila. Autophagy 2021:1-15.
7. Kern JV, Zhang YV, Kramer S, Brenman JE, Rasse TM. The kinesin-3, unc-104 regulates dendrite morphogenesis and synaptic development in Drosophila. Genetics 2013;195(1):59-72.
8. Zhu JY, Vereshchagina N, Sreekumar V, et al. Knockdown of Hsc70-5/mortalin induces loss of synaptic mitochondria in a Drosophila Parkinson's disease model. PLoS One 2013;8(12):e83714.
9. Butzlaff M, Hannan SB, Karsten P, et al. Impaired retrograde transport by the Dynein/Dynactin complex contributes to Tau-induced toxicity. Human molecular genetics 2015;24(13):3623-3637.
