# Supplementary material for: Bi-allelic loss-of-function variants in BCAS3 cause a syndromic neurodevelopmental disorder
Source: Am J Hum Genet. 2021 May 21;108(6):1069–82. doi: 10.1016/j.ajhg.2021.04.024 (PMC8206390; doi:10.1016/j.ajhg.2021.04.024)
Supplement: Document S2. Article plus supplemental information [file mmc8.pdf]

Bi-allelic loss-of-function variants in *BCAS3* cause a syndromic neurodevelopmental disorder

Holger Hengel,[1,2] Shabab B. Hannan,[1,2] Sarah Dyack,[3] Sara B. MacKay,[4] Ulrich Schatz,[5,6] Martin Fleger,[7] Andreas Kurringer,[7] Ghassan Balousha,[8] Zaid Ghanim,[9] Fowzan S. Alkuraya,[10,11] Hamad Alzaidan,[12] Hessa S. Alsaif,[10] Tadahiro Mitani,[13] Sevcan Bozdogan,[16] Davut Pehlivan,[13,14] James R. Lupski,[13,14,15] Joseph J. Gleeson,[17] Mohammadreza Dehghani,[18] Mohammad Y.V. Mehrjardi,[19] Elliott H. Sherr,[20] Kendall C. Parks,[20] Emanuela Argilli,[20] Amber Begtrup,[21] Hamid Galehdari,[22] Osama Balousha,[23] Gholamreza Shariati,[24] Neda Mazaheri,[25] Reza A. Malamiri,[26] Alistair T. Pagnamenta,[27] Helen Kingston,[28] Siddharth Banka,[28,29] Adam Jackson,[29] Mathew Osmond,[30] Care4Rare Canada Consortium,[30] Genomics England Research Consortium,[31] Angelika Rieß,[32] Tobias B. Haack,[32] Thomas Nägele,[34] Stefanie Schuster,[1,2] Stefan Hauser,[1,2] Jakob Admard,[32,33] Nicolas Casadei,[32,33] Ana Velic,[35] Boris Macek,[35] Stephan Ossowski,[32,33] Henry Houlden,[36] Reza Maroofian,[36,37,*] and Ludger Schöls[1,2,37,*]

Summary

BCAS3 microtubule-associated cell migration factor (BCAS3) is a large, highly conserved cytoskeletal protein previously proposed to be critical in angiogenesis and implicated in human embryogenesis and tumorigenesis. Here, we established *BCAS3* loss-of-function variants as causative for a neurodevelopmental disorder. We report 15 individuals from eight unrelated families with germline bi-allelic loss-of-function variants in *BCAS3*. All probands share a global developmental delay accompanied by pyramidal tract involvement, microcephaly, short stature, strabismus, dysmorphic facial features, and seizures. The human phenotype is less severe compared with the *Bcas3* knockout mouse model and cannot be explained by angiogenic defects alone. Consistent with being loss-of-function alleles, we observed absence of BCAS3 in probands' primary fibroblasts. By comparing the transcriptomic and proteomic data based on probands' fibroblasts with those of the knockout mouse model, we identified similar dysregulated pathways resulting from over-representation analysis, while the dysregulation of some proposed key interactors could not be confirmed. Together with the results from a tissue-specific *Drosophila* loss-of-function model, we demonstrate a vital role for BCAS3 in neural tissue development.

Introduction

BCAS3 microtubule-associated cell migration factor (BCAS3) is a large 928 amino acid, 101 kDa protein encoded by a 25-exon gene, *BCAS3* (MIM: 607470), that spans a genomic interval of 714 kb on chromosome 17q23.2. This highly conserved cytoskeletal protein is involved in human embryogenesis as well as in

[1]Department of Neurology and Hertie-Institute for Clinical Brain Research, University of Tübingen, 72076 Tübingen, Germany; [2]German Center of Neurodegenerative Diseases, 72076 Tübingen, Germany; [3]Division of Medical Genetics, Department of Pediatrics, Dalhousie University, Halifax, NS B3K 6R8, Canada; [4]Maritime Medical Genetics Service IWK Health Centre, Halifax, NS B3R 6R8 Canada; [5]Institute of Human Genetics, Medical University of Innsbruck, Peter-Mayr. Str. 1, 6020 Innsbruck, Austria; [6]Institute of Human Genetics, Technical University of Munich, Trogerstr. 32, 81675 Munich, Germany; [7]Department of Pediatrics, Landeskrankenhaus Bregenz, Carl-Pedenz-Str. 2, 6900 Bregenz, Austria; [8]Department of Pathology and Histology, Al-Quds University, Eastern Jerusalem 19356, Palestine; [9]Palestine Medical Complex, Ramallah, Palestine; [10]Department of Translational Genomics, Center for Genomic Medicine, King Faisal Specialist Hospital and Research Center, Riyadh 11211, Saudi Arabia; [11]Department of Anatomy and Cell Biology, College of Medicine, Alfaisal University, Riyadh 11533, Saudi Arabia; [12]Department of Medical Genetics, King Faisal Specialist Hospital and Research Center, Riyadh 11564, Saudi Arabia; [13]Department of Molecular and Human Genetics, Baylor College of Medicine, Houston, TX 77030, USA; [14]Department of Pediatrics, Baylor College of Medicine, Houston, TX 77030, USA; [15]Texas Children's Hospital, Houston, TX 77030, USA; [16]Department of Medical Genetics, Cukurova University Faculty of Medicine, 01330 Adana, Turkey; [17]Laboratory for Pediatric Brain Disease, Howard Hughes Medical Institute, Department of Neurosciences, University of California, San Diego, La Jolla, CA 92093, USA; [18]Medical Genetics Research Center, Shahid Sadoughi University of Medical Sciences, Yazd, Iran; [19]Abortion Research Centre, Yazd Reproductive Sciences Institute, Shahid Sadoughi University of Medical Sciences, Yazd, Iran; [20]Department of Neurology and Institute of Human Genetics and Weill Institute for Neurosciences, University of California, San Francisco, San Francisco, CA 94158, USA; [21]GeneDx, 207 Perry Parkway, Gaithersburg, MD 20877, USA; [22]Department of Genetics, Faculty of Science, Shahid Chamran University of Ahvaz, Ahvaz, Iran; [23]Faculty of Medicine, Al-Quds University, Eastern Jerusalem 19356, Palestine; [24]Department of Medical Genetics, Faculty of Medicine, Ahvaz Jundishapur University of Medical Sciences, Ahvaz, Iran; [25]Department of Genetics, Faculty of Science, Shahid Chamran University of Ahvaz, Ahvaz, Iran; [26]Department of Paediatric Neurology, Golestan Medical, Educational, and Research Center, Ahvaz Jundishapur University of Medical Sciences, Ahvaz, Iran; [27]National Institute for Health Research Oxford Biomedical Research Centre, Wellcome Centre for Human Genetics, University of Oxford, Oxford OX3 7BN, UK; [28]Manchester Centre for Genomic Medicine, St Mary's Hospital, Manchester University NHS Foundation Trust, Health Innovation Manchester, Manchester M13 9WL, UK; [29]Division of Evolution and Genomic Sciences, School of Biological Sciences, Faculty of Biology, Medicine, and Health, University of Manchester, Manchester M13 9WL, UK; [30]Children's Hospital of Eastern Ontario Research Institute, University of Ottawa, Ottawa, ON K1H 8L1, Canada; [31]Genomics England, London EC1M 6BQ, UK; [32]Institute of Medical Genetics and Applied Genomics, University of Tübingen, 72076 Tübingen, Germany; [33]NGS Competence Center Tübingen, University of Tübingen, 72076 Tübingen, Germany; [34]Department of Neuroradiology, University Hospital of Tuebingen, 72076 Tübingen, Germany; [35]Proteome Center Tübingen, University of Tübingen, 72076 Tübingen, Germany; [36]Department of Neuromuscular Disorders, Queen Square Institute of Neurology, University College London, London WC1N 3BG, UK
[37]These authors contributed equally
*Correspondence: rmaroofian@gmail.com (R.M.), ludger.schoels@uni-tuebingen.de (L.S.)
https://doi.org/10.1016/j.ajhg.2021.04.024.
© 2021 The Authors. This is an open access article under the CC BY license (http://creativecommons.org/licenses/by/4.0/).

tumorigenesis.[1] The mouse ortholog, *Bcas3,* is 98% identical to *BCAS3* and is essential for mouse development and angiogenesis.[2] Homozygous *Bcas3*$^{-/-}$ knockout leads to embryonic lethality in mice. Mutant embryos are growth retarded and show defects in the morphology and vasculature of the head and heart.[2] BCAS3 is well established as a critical protein regulating the cytoskeleton in endothelial migration as well as in sprouting angiogenesis. In human disease, *BCAS3* was found to be overexpressed in breast cancer[3] (hence, the previous gene name, breast carcinoma-amplified sequence 3), and high levels of BCAS3 were noted in tumor cells and blood vessels of different brain tumors, such as glioblastoma and hemangiopericytoma, as well as in brain abscesses.[1] Genome-wide association studies (GWASs) have associated variants in *BCAS3* with coronary artery disease[4] as well as with gout.[5] One previous study suggested rare homozygous missense variants in *BCAS3* as candidate variants for autosomal recessive intellectual disability.[6]

Here, we establish bi-allelic *BCAS3* variants as a cause of autosomal recessive syndromic global developmental delay. We used comparative transcriptomics and proteomics data from probands' fibroblasts to experimentally explore dysregulated pathways. While there are similarities to previous murine knockout studies on a global level, levels of several proposed key proteins, including CDC42 and Vimentin, were unchanged in probands' fibroblasts. Furthermore, there were no signs of defective angiogenesis in any of the identified probands, and migration assays using probands' primary fibroblasts did not show measurable defects in cell migration. We further explored the biological consequences of *BCAS3* dysfunction by investigating the phenotypes of a *Drosophila* loss-of-function model and confirmed an essential role of *rudhira* during development independent of angiogenesis.

Material and methods

Ethical approval

Written informed consent was obtained from the parents of the underage probands for diagnostic procedures and next-generation sequencing as well as for the publication of identifying facial images. This study was approved by the local institutional review board of the Medical Faculty of the University of Tübingen, Germany (vote 180/2010BO1).

Next-generation sequencing and analysis

Whole-exome or -genome sequencing was performed at different genetic institutes via next-generation sequencing techniques according to the local standard protocols. All variants were confirmed via Sanger sequencing with standard methods and chemicals (primer sequences are available upon request).

Family 1 and family 6

Exome sequencing of affected probands and data analysis were performed as previously described.[7]

Family 2

Exome sequencing for two affected siblings (F2-II.2 and F2-II.3) was performed at the Institute of Medical Genetics and Applied Genomics in Tübingen as previously described.[8] After excluding pathogenic or likely pathogenic variants in genes known to be associated with neurological or developmental disorders, variants were filtered for rare (gnomAD minor allele frequency $< 0.1\%$) homozygous or compound heterozygous variants shared between the two affected siblings.

Family 3

Exome sequencing was performed for the index proband in a CLIA-certified and CAP- and ISO 15189-accredited laboratory (Blueprint Genetics) as previously described.[9] Segregation of the identified variant was performed by Sanger sequencing in the Clinical Genomics Laboratory at IWK Health.

Family 4

Single-exome sequencing was performed on F4-II.1 as previously described.[10]

Family 5

Exome sequencing was performed as previously described.[11] The *BCAS3* variant was identified by sharing candidate genes among collaborators.

Family 7

Informed consent was provided according to the Baylor-Hopkins Center for Mendelian Genomics Research Protocol (IRB number: H-29697). Exome sequencing for two affected siblings (F7-II.1 and F7-II.2) was performed as previously described.[12]

Family 8

The affected proband and his unaffected parents were recruited to the 100K Genomes Project (100KGP),[13] a national genome sequencing initiative approved by the Health Research Authority Committee East of England, Cambridge South (REC: 14/EE/1112). Library preparation was performed with TruSeq DNA PCR-Free Library Prep, and sequencing was performed on a HiSeq X instrument. Variants were called jointly (as a trio) with Platypus v.0.7.9.5 and then filtered with the Genomics England Tiering process. We used Manta[14] to detect structural variants and called the paternally inherited 311 kb deletion that was previously detected via chromosomal microarray analysis (OGT 8x60k with CytoSure Interpret v.3.4.3.).

Family 9

Trio whole-exome sequencing (WES) of the affected proband and her parents was performed and analyzed by GeneDX as previously described.[15]

Fibroblast cultivation

Human dermal fibroblasts were maintained at 37°C, 5% CO_2, and 100% relative humidity in fibroblast medium consisting of Dulbecco's modified Eagle's medium (DMEM) (Merck) supplemented with 10% fetal bovine serum (FBS) (Thermo Fisher Scientific) in cell culture flasks. Cells were split via trypsinization and passaged into new flasks or seeded at a defined density for the Oris cell migration assay.

Oris cell migration assay

Fibroblast migration was investigated with an Oris cell migration plate (Platypus Technologies). Control (four lines) and proband fibroblasts (two lines) were isolated and seeded at a density of 6×10^4 cells per well into a 96-well Oris cell migration plate according to the manufacturer's guidelines (5 wells per cell line). After a pre-incubation step of 24 h, stoppers were removed and further incubated for 30 h. Afterward, the cells were stained with ActinRed 555 (Sigma) according to the manufacturer's instructions and imaged

with an Operetta High Content Imaging System (Perkin Elmer). Using ImageJ, we calculated the covered area per well.

Protein isolation and immunoblotting

Upon reaching high confluence, primary fibroblasts were washed with cold PBS, scraped off in PBS, centrifuged at 800 g for 5 min, and frozen at −80°C. Pellets of primary fibroblasts were lysed in RIPA buffer (Sigma) containing protease inhibitors (Roche) for 45 min on a rotator at 4°C. Cell debris were pelleted at 15,800 g and 4°C for 30 min. The protein concentration was determined with the Pierce BCA Protein Assay Kit (Thermo Fisher Scientific) according to the manufacturer's instructions. 10 μg of protein was eluted in 5× pink buffer (Thermo Fisher Scientific) at 95°C. Samples were separated on 10% polyacrylamide gels and transferred onto a Hypond-P polyvinylidene difluoride (PVDF) membrane (Merck). Membranes were blocked in 5% milk in TBS-T and incubated overnight with the primary antibodies against rabbit α-BCAS3 (1:1,000, Bethyl Laboratories), rabbit α-CDC42 (1:5,000, Abcam), rabbit α-Vimentin (1:5,000, Cell Signaling Technologies), and mouse α-GAPDH (1:20,000, Meridian Life Sciences) in Western Blocking Reagent (Roche) at 4°C, followed by three washes with TBS-T and incubation with HRP-conjugated secondary antibodies (Jackson ImmunoResearch) for 1 h at room temperature. Proteins were visualized with the Immobilon Western chemiluminescent HRP substrate (Merck).

RNA isolation and RNA sequencing

For RNA extraction, fibroblasts from two primary proband fibroblast cell lines (F3-II.1 and F4-II.1) and two primary control fibroblast cell lines (CO-1, female, 24 years old and CO-2, male, 22 years old) were cultivated. RNA was isolated from three independently grown flasks for each cell line (biological replicates, 4 × 3 RNA samples) via the QIAGEN RNeasy Mini Kit. RNA quality was assessed with the Agilent 2100 Bioanalyzer RNA Nano Kit (Agilent Technologies, Santa Clara, CA, United States). All samples had high RNA integrity numbers (RIN > 9). Using the NEBNext Ultra II Directional RNA Library Prep Kit (New England Biolabs, Ipswich, MA, United States) with 100 ng of total RNA input for each sequencing library, we generated poly(A)$^{+}$-selected sequencing libraries according to the manufacturer's manual. All libraries were sequenced on the Illumina NovaSeq 6000 platform in paired-end mode with 2 × 51 bp reads and at a depth of approximately 30 million clusters each. Library preparation and sequencing procedures were performed by the same individual, and a design aimed to minimize the introduction of technical batch effects was chosen.

We assessed the quality of raw RNA sequencing (RNA-seq) data in FASTQ files by using ReadQC (v.2019_11) to identify potential sequencing cycles with low average quality and base distribution bias. Reads were preprocessed with SeqPurge (v.2019_11) and aligned with STAR (v.2.7.3a), allowing spliced read alignment to the human reference genome (build GRCh37). Alignment quality was analyzed with MappingQC (ngs-bits v.2019_11) and visually inspected with the Broad Integrative Genome Viewer (v.2.7.0). On the basis of the Ensembl genome annotation (GRCh37, Ensembl release 97), we obtained read counts for all genes by using subread (v.2.0.0).

For gene expression analysis, we filtered raw gene read counts to retain only genes with at least 1 count per million (cpm) in at least half of the samples and normalized them by the trimmed mean of M values (TMM) procedure, leaving >13,000 genes for determining differential expression in each of the pairwise comparisons between affected and control samples. The analysis was performed with edgeR (v.3.26.8) with negative binomial distributions and genewise testing with generalized linear models.

For each proband, triplicate expression profiles from corresponding fibroblast cell lines were compared to the average of both sets of triplicate control cell lines. In addition, the average of both proband triplicate sets was compared to the average of control triplicate sets (Table S2).

Gene expression changes are expressed as log2-fold changes and expression in the control group was used as a baseline.

Significant genes with an adjusted p value (false discovery rate [FDR]) of less than 0.01 and an absolute log2-fold change of at least 1 are reported.

NanoLC-MS/MS analysis and data processing

Protein samples for liquid chromatography-mass spectrometry (nanoLC-MS/MS) were generated from the two primary proband fibroblast cell lines in biological replicates (F3-II.1 and F4-II.1 corresponding to label-free quantification [LFQ] intensity proband [pat]_1, 2, 3 and LFQ intensity pat_4, 5, 6 in Table S4) and compared to three primary control fibroblast cell lines (LFQ intensity control [ctrl]_1, 2, 3). Protein extracts were purified with SDS-PAGE (Invitrogen). Coomassie-stained gel pieces were excised and in-gel digested via trypsin as described previously.[16] After desalting with C18 stage tips,[17] extracted peptides were separated on an Easy-nLC 1200 system coupled to a Q Exactive HF mass spectrometer (both Thermo Fisher Scientific) as described previously[18] with slight modifications. The peptide mixtures were separated via a 2-h segmented gradient from 10%–33%–50%–90% HPLC solvent B (80% acetonitrile in 0.1% formic acid) in HPLC solvent A (0.1% formic acid) at a flow rate of 200 nL/min. The 12 most intense precursor ions were sequentially fragmented in each scan cycle via higher energy collisional dissociation (HCD) fragmentation. In all measurements, sequenced precursor masses were excluded from further selection for 30 s. The target values for MS/MS fragmentation were 10^5 charges and 3×10^6 charges for the MS scan.

Acquired MS spectra were processed with the MaxQuant software package v.1.5.2.8[19] with an integrated Andromeda search engine.[20] A database search was performed against a target-decoy *Homo sapiens* database obtained from UniProt, containing 96,817 protein entries and 286 commonly observed contaminants. Endoprotease trypsin was defined as protease with a maximum of two missed cleavages. Oxidation of methionine and N-terminal acetylation were specified as variable modifications, whereas carbamidomethylation on cysteine was set as a fixed modification. The initial maximum allowed mass tolerance was set to 4.5 parts per million (ppm) for precursor ions and 20 ppm for fragment ions. Peptide, protein, and modification site identifications were reported at an FDR of 0.01, estimated by the target/decoy approach.[21] The LFQ algorithm was enabled, and matches between runs[22,23] and LFQ protein intensities were used for relative protein quantification. Downstream bioinformatics analyses (two-sample t tests and volcano plots) were performed with the Perseus software package v.1.5.0.15. Data were filtered for contaminants and reverse sequences and were only identified by site entries.

Over-representation analysis

Ontology and pathway analyses for MS data as well as RNA-seq data were performed with the WEB-based gene set analysis toolkit

2019 (WebGestalt).[24] For over-representation analysis (ORA), we used the Gene Ontology database and selected the "Biological Process noRedundant" category. As an initial gene list, significant up- or downregulated genes from the RNA-seq data (Table S3) or proteins from the MS data (Table S4) were used. As a reference gene list, all identified genes/proteins from RNA-seq and MS data, respectively, were used.

Drosophila husbandry and strains

Both control and *rudhira* knockdown crosses were maintained in Instant *Drosophila* Medium (Carolina). Crosses were set up at 25°C or 29°C and kept in incubators with a 12-h day-night cycle. The following lines were utilized in this study: two UAS-RNAi lines against *rudhira*, UAS-*rudhira*-RNAi-a (Bloomington *Drosophila* Stock Center [BDSC] #51691, *rudhira* RNAi inserted on 3rd chromosome) and UAS-*rudhira*-RNAi-b (Vienna *Drosophila* Resource Center [VDRC ID dna9673]); GAL4 driver lines *elav*-Gal4 (BDSC #8765, BDSC #5144), *D42*-Gal4 (BDSC #8816), and *Appl*-Gal4 (from Aaron Voigt, Department of Neurology in RWTH Aachen University); control lines for rudhira-RNAi-a (BDSC #36304, TRiP RNAi background lines with attP40 site on 3rd chromosome); and UAS-*GFP*-RNAi (BDSC #9331, *GFP* RNAi inserted on 2nd chromosome) as second RNAi control line. To generate the transgenic UAS-*rudhira*-RNAi-b *Drosophila* line, we diluted the construct received from VDRC (1 μL of an ~50–100 ng/μL stock) by adding 10 μL of TE buffer and mixed thoroughly. DH5α-competent cells were transformed with 2 μL from the diluted mixture. Agar plates containing ampicillin were plated and incubated overnight at 37°C. Individual colonies were picked and inoculated overnight in liquid culture followed by plasmid purification. Purified DNA was sent to Bestgene (BestGene, CA, United States) for embryo microinjection.

Results

Bi-allelic loss-of-function variants in *BCAS3* cause a syndromic neurodevelopmental disorder

To unravel the genetic cause of a neurodevelopmental disorder, we independently investigated affected probands from the consanguineous families F1, F2, and F3 (Figure 1) by using WES. The molecular diagnostic analysis did not show pathogenic or likely pathogenic variants in genes known to be associated with neurological or developmental disorders in any of these families. WES data were next screened for potential candidate variants, including heterozygous, compound heterozygous, and homozygous variants. Independently, in all three families, predicted homozygous loss-of-function variants in BCAS3 microtubule-associated cell migration factor (*BCAS3*) were identified, namely, the homozygous stop-gain variant c.73C>T (p.Gln25*) (GenBank: NM_001099432.3) in *BCAS3* in F1 and the homozygous variants c.726T>G (p.Tyr242*) and c.1457C>G (p.Ser486*) in F2 and F3, respectively. All three variants were absent from in-house databases as well as from public databases (not present in gnomAD v.2.1.1 or gnomAD v.3).[25] Furthermore, there were no homozygous *BCAS3* loss-of-function variants present in gnomAD, and a low observed/expected (o/e) ratio of 0.31 (0.22–0.47) hints at an intolerance for *BCAS3* loss-of-function variants. Sanger sequencing independently confirmed the variant alleles that segregated in accordance with Mendelian expectations in all available healthy and affected family members (Figure 1A).

Using the GeneMatcher platform,[26] we matched these three independent index families. A similar syndromic phenotype in all probands confirmed *BCAS3* loss-of-function variants as responsible for an autosomal recessive neurodevelopmental disorder. We next screened the GENESIS database, Solve-RD database, Munich Exome database (EVAdB), Baylor-Hopkins Center for Mendelian Genomics (BH-CMG) database, 100KGP, GeneDx, and Queen Square Genomics database for additional *BCAS3* probands to strengthen the evidence for this genotype-phenotype association. In this way, we were able to identify five additional families with a neurodevelopmental phenotype and bi-allelic *BCAS3* variants. In total, 15 affected individuals from eight families were studied (Figure 1A), including two families with compound heterozygous variants and six families with homozygous variants, the latter as anticipated given historical reports of consanguinity in the majority of families. All variants were absent or very rare in public databases (minor allele frequency [MAF] < 0.01% in gnomAD). The bi-allelic heterozygous missense variants identified in F4-II.1 were in highly conserved residues (phyloP 100-way of 7.49 and 9.48) and received high *in silico* prediction scores (CADD scores of 26.4 and 24.1) (Figure 1B, Table S1). In F8-II.1, chromosomal microarray analysis combined with trio genome sequencing led to the identification of a paternally inherited intragenic 311 kb deletion spanning exons 13 to 23 of *BCAS3* (chr17: g.60921177_61232193del [c.994−3230_2426−136134del]) and a maternally inherited near splice-site deletion of 4 bases (c.2074+4_2074+7delAGTA). The 311 kb deletion was detected by microarray analysis as well as in the genomes of the proband and his father and was confirmed via Sanger sequencing. Breakpoint locations and a microhomology with two bases are evidence of an *Alu*-LINE (long interspersed nuclear element) rearrangement[27] as mechanism (Figure S1).

All 15 probands harboring bi-allelic variants in *BCAS3* presented with a characteristic core phenotype consisting of severe global developmental delay (GDD), pyramidal tract involvement, microcephaly, and short stature (Table 1, Figure 2A). At the time of examination, probands were between 19 months and 19 years of age. All probands had severe GDD and intellectual disability (ID) (F3-II.2 was too young to be formally diagnosed with ID). Ten probands had a minimal vocabulary (between two and 12 words), and four never learned to speak. All probands had a severe motor disorder with pyramidal tract involvement resulting in hyperreflexia and spasticity of the lower limbs (15/15), while some probands had additional dystonic or dyskinetic movements (5/15). Ten of 14 probands achieved the ability to walk, although this milestone was significantly delayed. Regression of motor abilities starting between the ages of 10 and 15 years was reported for older probands. Seizures were observed in 7 of 15 probands—two probands had febrile

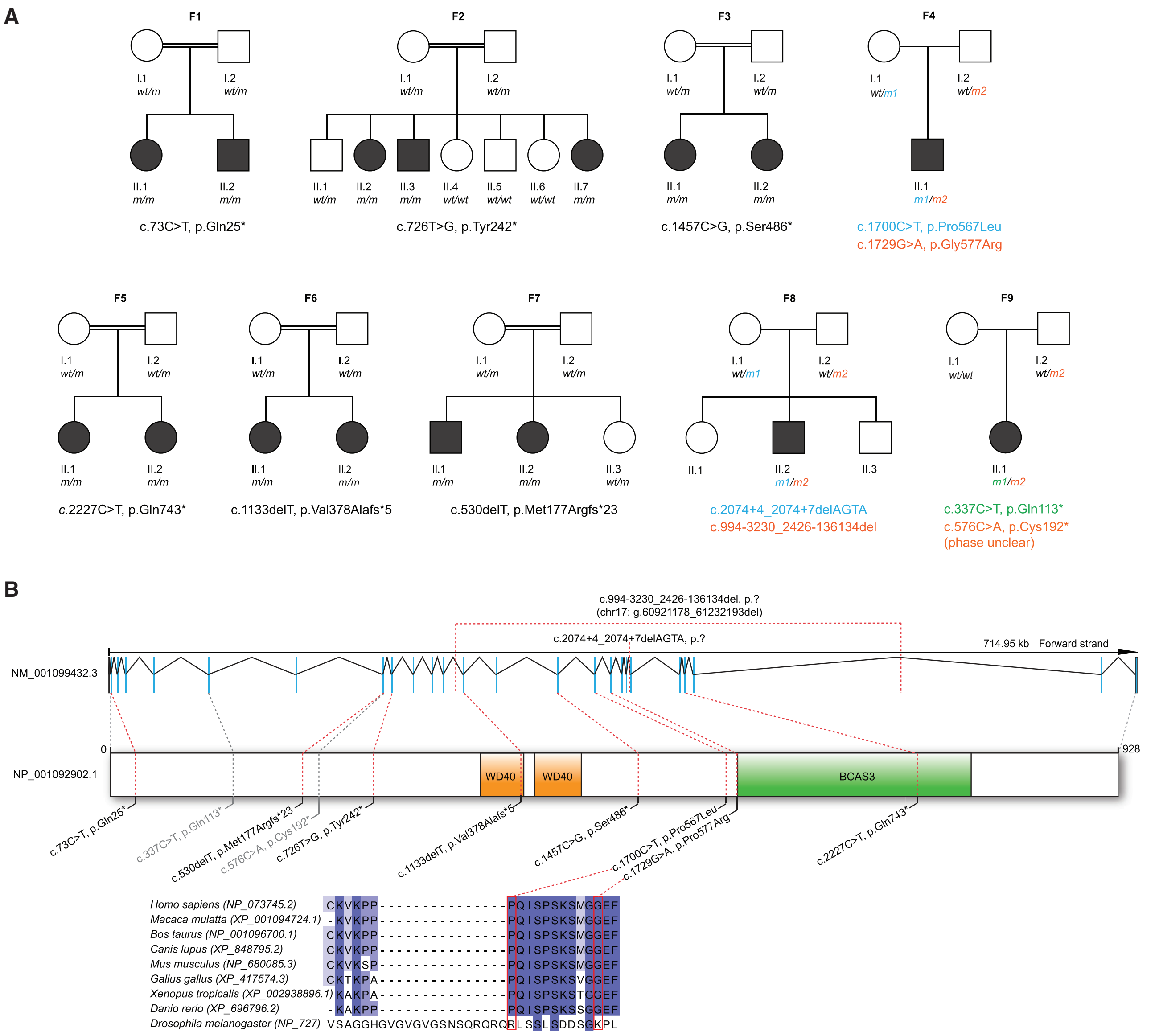

Figure 1. Pedigrees and genetic variants

(A) Pedigrees of eight families segregating syndromic GDD. Filled black symbols indicate affected individuals. Variants in *BCAS3* are presented below the pedigrees. Homozygous variants are presented as *m/m* in the pedigree. Compound heterozygous variants are presented with *m1* (cyan) and *m2* (orange). For F9, the phase of the *BCAS3* variants was unclear.

(B) *BCAS3* genomic and protein domain structures. Type and position of ten identified germline variants. Two additional variants from F9 were depicted in gray because the phase was not shown as in the other variants. Conservation across species is shown for the positions of the two compound heterozygous missense variants.

convulsions and five probands had generalized tonic or tonic-clonic seizures. Only one of these probands had pharmaco-resistant epilepsy.

All probands were born with length, weight, and head circumferences within normal ranges. Eleven of 13 probands developed microcephaly (median −3.1 SD), and 11 of 14 probands developed a short stature (median −2.6 SD). Two probands were normocephalic at the time of examination (F3-II.1, −0.58 SD and F3-II.2, −1.23 SD); however, they may still develop mild microcephaly, as they were 5 years and 19 months of age, respectively, at the time of examination. Minor dysmorphic facial features were observed in most probands, including full lips (9/12) with an everted lower lip (15/15); a short philtrum (8/11); large malformed, wide-spaced, or mispositioned teeth (10/14); prominent (10/15) and curved (11/15) eyebrows; an open mouth (15/15); and a long face (13/15) (Figure 2B, Table 1). Another frequent feature was strabismus, present in ten of 15 probands. The results of cardiovascular examinations, including transthoracic echocardiography, were mostly unremarkable. An asymptomatic bicuspid aortic valve was detected in two probands. Brain magnetic resonance images (MRIs) of 13 probands revealed a thin dysgenic corpus callosum, mostly

Table 1. Clinical and genetic findings in individuals with bi-allelic *BCAS3* variants

	F1-II.1	F1-II.2	F2-II.2	F2-II.3	F2-II.7	F3-II.1	F3-II.2	F4-II.1	F5-II.1	F5-II.2	F6-II.1	F6-II.2	F7-II.1	F7-II.2	F8-II.2	F9-II.1
**cDNA (GenBank:** NM_001099432.3**)**	c.73C>T, hom	c.73C>T, hom	c.726T>G , hom	c.726T>G , hom	c.726T>G , hom	c.1457C>G, hom	c.1457C>G, hom	c.[1700C>T];[1729G>A]	c.2227C>T, hom	c.2227C>T, hom	c.1133delT, hom	c.1133delT, hom	c.530delT, hom	c.530delT, hom	c.2074+4_2074+7delAGTA; intragentic deletion exon 13–23	c.[576C>A];[337C>T] (phase unclear)
**Protein (GenBank:** NP_001092902.1**)**	p.Gln25*, hom	p.Gln25*, hom	p.Tyr242*, hom	p.Tyr242*, hom	p.Tyr242*, hom	p.Ser486*, hom	p.Ser486*, hom	p.[Pro567Leu];[Gly577Arg]	p.Gln743*, hom	p.Gln743*, hom	p.Val378 Alafs*5, hom	p.Val378 Alafs*5, hom	p.Met177 Argfs*23	p.Met177 Argfs*23	p.[?]; [?]	p.[Cys192*];[Gln113*]
**Sex**	male	female	female	male	female	female	female	male	female	female	female	female	male	female	male	female
**Ethnic background**	Iranian	Iranian	Arab-Palestine	Arab-Palestine	Arab-Palestine	European descent	European descent	Albanian	Saudi	Saudi	Iranian-Lor	Iranian-Lor	Turkish	Turkish	European descent	European-Chinese
**Age (years) at examination**	19	13	19	18	5	5	19 months	8	15	11	11	6	7	6	18	14
**Current height (cm)**	160 (−2.3 SD)	152 (−0.7 SD)	136 (−4.2 SD)	142 (−4.4 SD)	95 (−2.8 SD)	114 (+1.28 SD)	78 (−0.92 SD)	109 (−3.3 SD)	140 (−3.4 SD)	129 (−2.1 SD)	127 (−2.4 SD)	90 (−5.0 SD)	110 (−2.2 SD)	96 (−3.8 SD)	N/A	147.5 (−1.37 SD)
**Current weight (kg)**	44 (−3.0 SD)	34 (−1.7 SD)	42 (−8.0 SD)	33 (−3.9 SD)	25 (+1.9 SD)	10 (+1.39 SD)	10 (−1.11 SD)	35 (+1.5 SD)	46 (−0.9 SD)	23 (−6.3 SD)	20 (−3.1 SD)	14 (−2.7 SD)	17 (−2.3 SD)	13 (−3.2 SD)	40.4 (−3.0 SD)	N/A
**Current head circumference (cm)**	51 (−2.8 SD)	49.5 (−3.2 SD)	50.0 (−4.0 SD)	52.2 (−1.9 SD)	46.5 (−2.8 SD)	49.5 (−0.58 SD)	45 (−1.23 SD)	43.0 (−6.7 SD)	N/A	N/A	48 (−3.8 SD)	47 (−3.1 SD)	47 (−3.8 SD)	46 (−3.9 SD)	51 (age 15 years, −2.6 SD)	53 (−0.80 SD)
**Motor development**	sitting at 18 months, walking at 36 months	sitting at 17 months, walking at 36 months	sitting at 15 months, walking at 36 months	sitting at 17 months, walking at 72 months	sitting at 17 months, walking at 30 months	sitting at 13 months, walking at 30 months	sitting at 15 months, commando crawling 15 months	no sitting or walking	sitting at 10 months, walking at 33 months	sitting at 9 months, walking at 26 months	cannot walk	cannot walk	sitting at 1 year, walking at 4 years	sitting at 1 year, cannot walk	sitting at 1 year, walking at 4 years	sitting at 6 months, walking at 36 months
**Cognition at last follow-up**	severe ID	severe ID	severe ID	severe ID	severe ID	severe ID	severe GDD	severe ID	severe ID (IQ = 45)	severe ID (IQ = 55)	severe ID	severe ID	severe ID	severe ID	severe ID	severe ID
**Speech at last follow-up**	can speak, about ten words	can speak, about ten words	no speech	no speech	can speak two words	can speak about five words	babbles, no true words	no speech	can speak, about ten words	can speak, about ten words	can speak, about three words	can speak, about six words	can speak, about ten words	no speech	can speak, about nine words	can speak, about 12 words
**Epilepsy, seizure types, age of onset**	no	no	no	no	one febrile convulsion, 36 months	febrile convulsions about every 6 months, 29 months	no	GTCS, age of 4 months, pharmaco-responsive	two tonic seizures, 5 years	no	tonic seizures, 10 years, pharmaco-resistant	tonic seizures, 2 years, pharmaco-responsive	three febrile seizures, 6 months	no	no	complex partial seizures, 1–2 spells/day
**Neurological examination**	hyperreflexia, lower limb spasticity, spastic gait	hyperreflexia, lower limb spasticity, spastic gait	hyperreflexia, lower limb spasticity, feet contractures, spastic gait	hyperreflexia, lower limb spasticity, feet contractures, non-ambulatory	hyperreflexia, lower limb spasticity, spastic gait	hyperreflexia, lower limb spasticity	hyperreflexia, lower limb spasticity	hyperreflexia, lower limb spasticity, feet contractures, non-ambulatory	hyperreflexia, lower limb spasticity, dystonia, gait imbalance	hyperreflexia, lower limb spasticity, dystonia, gait imbalance	hyperreflexia, severe hypertonia, dyskinetic movements, non-ambulatory	hyperreflexia, severe hypertonia, dyskinetic movements, non-ambulatory	hyperreflexia, lower limb spasticity, mild contracture in knees, non-ambulatory (can crawl)	hyperreflexia, lower limb spasticity, mild contracture in knees, non-ambulatory (can crawl)	hyperreflexia, overall hypotonic but lower limb spasticity, dystonia	hyperreflexia, progressive spasticity, spastic quadriplegic

(*Continued on next page*)

Table 1. *Continued*

	F1-II.1	F1-II.2	F2-II.2	F2-II.3	F2-II.7	F3-II.1	F3-II.2	F4-II.1	F5-II.1	F5-II.2	F6-II.1	F6-II.2	F7-II.1	F7-II.2	F8-II.2	F9-II.1
**Dysmorphic facial features**	prominent full and curved eyebrows, synophrys, full lips, everted lower lip, short philtrum, wide-spaced teeth, open mouth, long face	prominent full and curved eyebrows, synophrys, full lips, everted lower lip, short philtrum, wide-spaced teeth, open mouth, long face	prominent full eyebrows, ptosis, synophrys, full lips, everted lower lip, short philtrum, large malpositioned teeth, open mouth, long face	prominent full eyebrows, ptosis, full lips, everted lower lip, short philtrum, large malpositioned teeth, open mouth, long face	prominent full eyebrows, synophrys, full lips, everted lower lip, short philtrum, open mouth, long face	curved eyebrows, synophrys, wide-spaced eyes, broad nasal bridge, full lips, open mouth, everted lower lip	epicanthic folds, full lips with everted lower lip, eyebrows sparse and light blonde	full lips, everted lower lip, short philtrum, large malpositioned teeth, open mouth, long face	prominent full and curved eyebrows, hypertelorism, broad nasal bridge, everted lower lip, open mouth, long face	curved eyebrows, hypertelorism, broad nasal bridge, everted lower lip, open mouth, long face	prominent full and curved eyebrows, synophrys, short philtrum, large spaced malpositioned teeth, full lips, everted lower lip, open mouth, long face	curved eyebrows, synophrys, large spaced malpositioned teeth, full lips, everted lower lip, open mouth, long face	prominent full and curved eyebrows, synophrys, short philtrum, everted lower lip, wide-spaced teeth, open mouth, long face	prominent full and curved eyebrows, synophrys, everted lower lip, wide-spaced teeth, open mouth, long face	prominent full and curved eyebrows, synophrys, everted lower lip, short philtrum, open mouth, long face	large mouth with poor dental work (maybe secondary)
**Instrumental examinations**																
**Ophthalmologic findings**	strabismus	Strabismus	strabismus, ptosis	strabismus, ptosis	strabismus, saccade initiation difficulties	strabismus	strabismus	strabismus, nystagmus, myopia	normal	normal	visual impairment, strabismus (extropia)	visual impairment, strabismus (extropia)	normal	normal	nystagmus	nystagmus, visual impairment
**Cardio-vascular (e.g., echo results)**	N/A	N/A	TTE: bicuspid aortic valve	TTE: bicuspid aortic valve	normal TTE	normal TTE	N/A	normal TTE	normal TTE	normal TTE	normal TTE	normal TTE	normal TTE	normal TTE	N/A	normal TTE
**Age, brain imaging**	9 years, posterior thin corpus callosum, impaired myelination	5 years, posterior thin corpus callosum, impaired myelination	22 years, posterior thin corpus callosum, cerebral and cerebellar atrophy, normal MRA	21 years, posterior thin corpus callosum, cerebral and cerebellar atrophy, normal MRA	7 years, posterior thin corpus callosum, cerebral and cerebellar atrophy, normal MRA	14 months, posterior thin corpus callosum, hyperintensity of dentate nuclei on T2	N/A	5 months, posterior thin corpus callosum, delayed myelination, hypoplastic pons and cerebellum	9 years, posterior thin corpus callosum, cerebellar atrophy, impaired myelination	4.5 years, posterior thin corpus callosum, cerebellar and cerebral atrophy, delayed myelination, VGAM	7 years, posterior thin corpus callosum, cerebral and cerebellar atrophy	4 years, posterior thin corpus callosum, delayed myelination, cerebral and cerebellar atrophy	6 years, posterior thin corpus callosum, impaired myelination	7 years, posterior thin corpus callosum, impaired myelination	N/A	11 years, posterior thin corpus callosum, diminished white matter volume

Abbreviations are as follows: GTCS, generalized tonic-clonic seizure; TTE, transthoracic echocardiogram; MRA, magnetic resonance angiography; VGAM, vein of Galen aneurysmal malformation; ID, intellectual disability; GDD, global developmental delay.

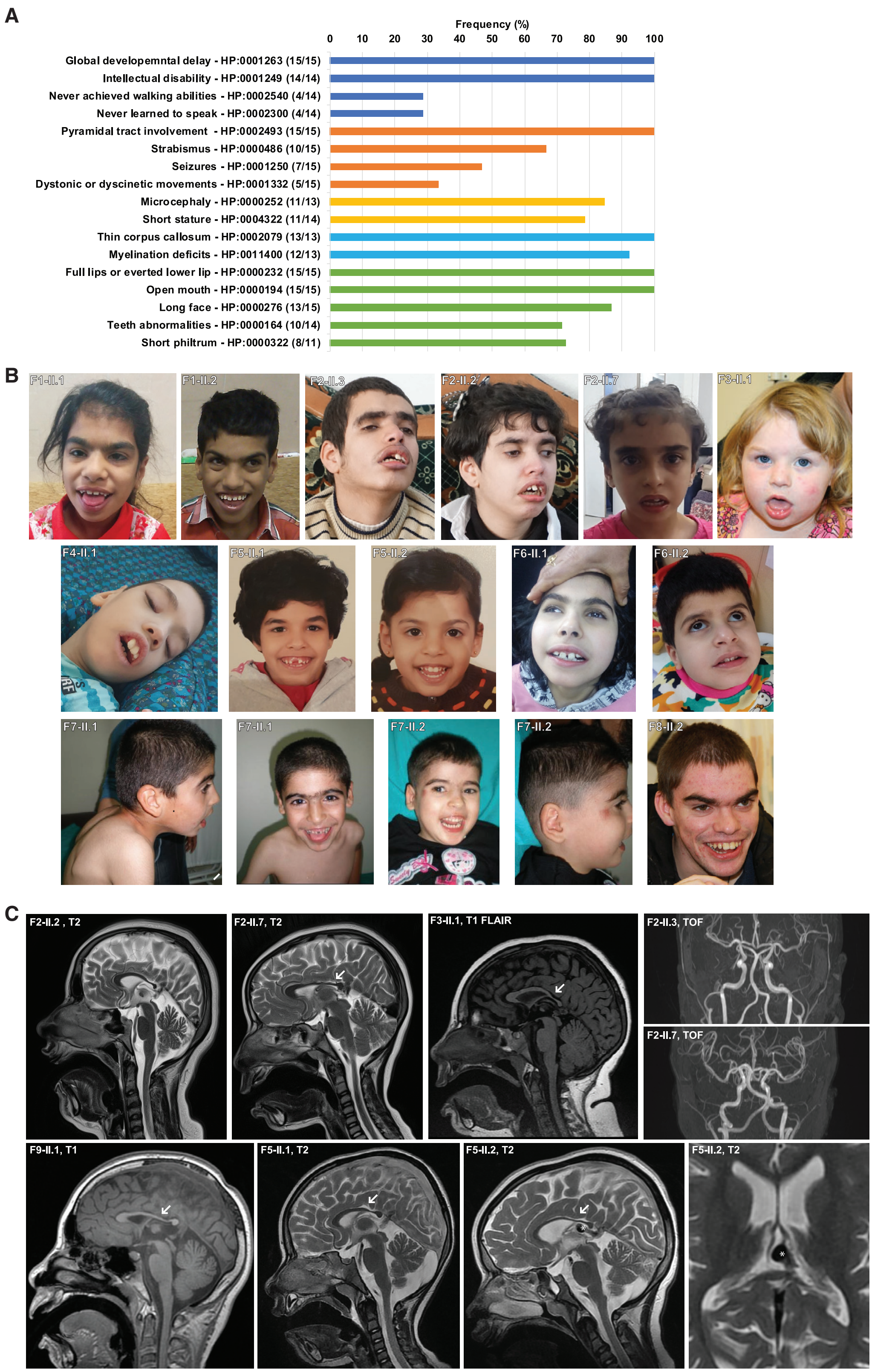

Figure 2. Phenotypic features of probands with bi-allelic *BCAS3* variants
(A) Prevalence of signs and symptoms in probands with bi-allelic *BCAS3* variants. The numerator and denominator in brackets indicate the number of affected probands and the number of probands assessed for the respective feature, respectively.
(B) Facial photographs of 14 affected individuals with mild dysmorphic facial features, including full lips with an everted lower lip; short philtrum; mispositioned, wide-spaced large teeth; and synophrys. Furthermore, ptosis is present in F1-II.1, F2-II.2, and F2-II.3, and strabismus can be seen in several photographs except for in those of F4-II.1, F5-II.1, F5-II.2, F7-II.1, F7-II.2, and F8-II.2.

(legend continued on next page)

affecting the splenium in all 13 probands. Furthermore, moderate cerebral and cerebellar atrophy (more pronounced in the older probands) and delayed myelination (observed in the younger probands) were present in most probands (Figure 2C, Table 1, Table S2). A vascular malformation, most likely a vein of Galen malformation, was identified in one proband (F5-II.2, Figure 2C). Otherwise, time-of-flight magnetic resonance angiography (TOF-MRA) in three probands did not reveal an abnormal cerebral blood vessel configuration.

In another additonal proband, F9-II.1, two *BCAS3* stop-gain variants (one paternal and the second *de novo*) were identified. Although the phase of the variants was not determined, we regarded a bi-allelic distribution most likely given the striking phenotypic similarities encompassing the characteristic core clinical features (Table 1).

BCAS3 is absent in proband-derived fibroblasts

To compare the protein levels of BCAS3, we performed immunoblotting analysis from fibroblasts of probands F3-II.1 and F4-II.1 and control cell lines. BCAS3 was undetectable in both proband-derived cell lines, while it was clearly detected in the control fibroblast lines (Figure 3A). Although we did not observe any full-size BCAS3, we cannot rule out the presence of a truncated protein in F3-II.1, as the homozygous stop-gain variant p.Ser486* lies upstream of the antibody detection site (position 870 to C terminus). More interestingly, BCAS3 was also not detectable in fibroblasts from F4-II.1, harboring two compound heterozygous missense variants (c.1700C>T [p.Pro567Leu] and c.1729G>A [p.Gly577Arg]). This indicates that the missense variants lead to a misfolded or unstable protein that is most likely degraded.

Exploring dysregulated pathways in proband-derived fibroblasts

Next, we examined two previously proposed interaction partners of BCAS3, namely, CDC42 and Vimentin, by using immunoblotting. Both were present at roughly estimated similar protein levels in proband fibroblasts and control fibroblast cell lines (Figure 3A). To further investigate the global impact of the bi-allelic *BCAS3* variants on different pathways, we performed RNA-seq and nanoLC-MS/MS comparing the two proband cell lines with healthy control cell lines. The transcriptomic data showed a significant reduction in *BCAS3* mRNA in F3-II.1 of −2.92 log fold change (logFC), indicating partial nonsense-mediated decay due to the homozygous nonsense variant, while the mRNA levels were not significantly reduced in F4-II.1 (−0.21 logFC).

With nanoLC-MS/MS, BCAS3 was not detectable in either proband cell line, consistent with the results from immunoblotting. However, BCAS3 was probably at the detection limit of this method, as nanoLC-MS/MS was able to detect BCAS3 in only two out of three controls, rendering immunoblotting the more reliable method for addressing this question. Next, we examined the correlation between transcriptomics and proteomics data. RNA-seq was able to detect mRNAs from 13,223 different genes. For 5,483 proteins, corresponding proteomics data from nanoLC-MS/MS were available. MS did not detect any proteins for which no RNA data were present. Expression changes with significant up- or downregulated genes at the mRNA level (FDR < 0.01) correlated very well with significant up- or downregulated proteins in MS (rho $= 0.89$, $p = 2.2 \times 10^{-16}$) (Figure S2A). The high correlation coefficient indicates high data quality and reliability. Similarly, proteins that could only be measured in controls (but not in probands) via MS were significantly downregulated in the RNA-seq data, while proteins that were only detectable in probands (but not in controls) via MS were upregulated in the RNA-seq data (Figure S2B). Following this proof of consistency, we analyzed expression of previously proposed key interaction partners of BCAS3, such as *ACTB*, *CDC42*, *PTK2*, *PTK2B*, *TGFB*, *TGFBR*, *VEGFA*, *VEGFR*, and *VIM*. Substantial mRNA expression changes (>0.5 |log FC|) were only present in PTK2B (−1 logFC) and vimentin (0.52 logFC) (Table S5). Corresponding MS data were only present for a few of these proteins and did not show any substantial changes at the protein level. Specifically, for PTK2B, there were no MS data available, and vimentin was not significantly changed.

We next used WebGestalt for ORA of RNA-seq and MS data. From the RNA-seq data, 804 genes were significantly up- or downregulated (FDR < 0.01 and absolute |log FC| $\geq$ 1.00, Table S3). The resulting ORA showed the following biological processes (Gene Ontology terms) as top hits (Figure 3B): "extracellular structure organization," "angiogenesis," and "epithelial cell proliferation." In the nanoLC-MS/MS data, 59 proteins were significantly up- or downregulated (log Student's t test p value ≤ -1.3 and absolute |log FC $\geq$ 1.00|, Table S4). ORA resulted in "extracellular structure organization" as a top hit followed by "angiogenesis" and "actomyosin structure organization" with a considerable margin (Figure 3C).

Migration of proband-derived fibroblasts is not affected

Because the promotion of cell migration is supposed to be one primary function of BCAS3 and was impaired in the knockout mouse model,[2] we investigated the migration of proband-derived fibroblasts. A cell migration assay using the two proband cell lines (F3-II.1 and F4-II.1) and four control fibroblast lines showed no impairment of migration in proband fibroblasts compared with control fibroblast lines (Figure 3D).

(C) Exemplary MRI of probands from families F2, F3, F5, and F9. Midsagittal T2/T1 showing a thin dysgenic corpus callosum (white arrow), especially of the posterior part (splenium). This is present in all available MRIs. Coronal TOF-MRA displaying normally developed large arterial blood vessels of the brain. A vascular malformation, most likely a vein of Galen malformation, was identified in one proband (F5-II.2) and is marked with a white asterisk on the T2 images.

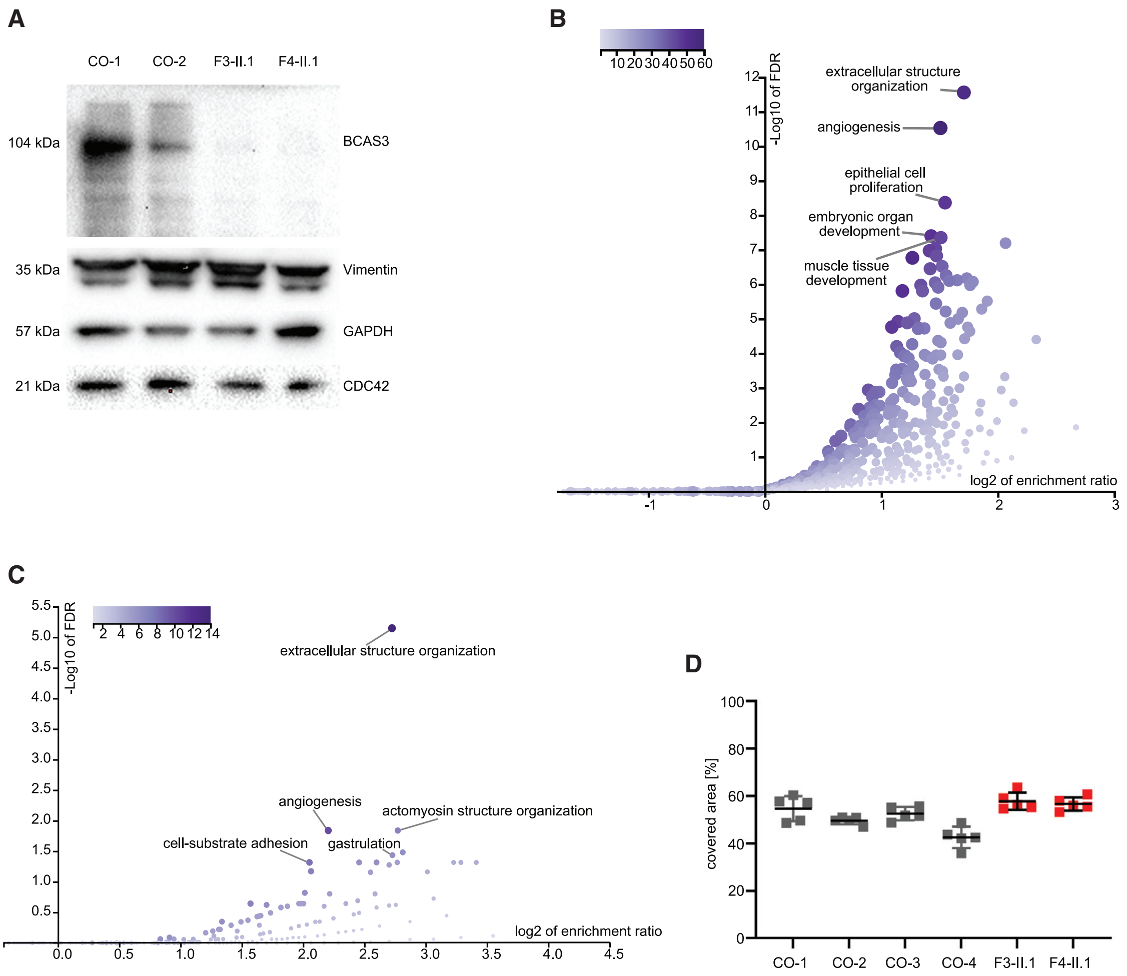

Figure 3. Analyses based on proband fibroblasts

(A) Protein analysis by immunoblotting comparing two proband cell lines (F3-II.1 and F4-II.1) and two control cell lines (CO-1 and CO-2). BCAS3 is absent from both proband cell lines. Vimentin and CDC42 were detected in roughly comparable levels in both probands and controls (no semiquantitative analysis was performed). GAPDH was used as a loading control.

(B) Volcano plot resulting from ORA for Gene Ontology biological processes (WebGestalt) based on significant up- or downregulated genes from the transcriptomic data (804 genes, Table S3). Purple color intensity indicates the number of overlapping genes per category.

(C) Volcano plot resulting from ORA for Gene Ontology biological processes (WebGestalt) based on significant up- or downregulated proteins from the proteomics data (59 proteins, Table S4).

(D) Assay comparing migration rates in control (CO-1 to CO-4) and proband (F3-II.1 and F4-II.1) fibroblasts. Five technical replicates from each cell line were tested (gray and red squares). A higher percentage of covered area indicates faster migration. The mean covered area was even slightly higher in proband fibroblasts compared to control fibroblasts (57.3% ± 3.2% [SD] and 49.9% ± 5.7%, respectively); $p < 0.001$. However, migration rates in CO-4 were significantly slower compared to the other three control cell lines and the two proband cell lines, which showed comparable migration rates.

rudhira silencing induces tissue-specific developmental defects in a *Drosophila* loss-of-function model

In mice, loss of *Bcas3* causes embryos to die on embryonic day (E) 9.5.[2] We used a *Drosophila* loss-of-function model to further investigate the role of *rudhira*, which shares 32% identity with human *BCAS3*, in tissue-specific development. Using the temperature-dependent UAS-Gal4 expression system,[28,29] we expressed two different RNAi constructs against *rudhira* (BDSC #51691 and VDRC ID dna9673, hereafter referred to as *rudhira*-RNAi-a and *rudhira*-RNAi-b, respectively) under different neuronal Gal4 drivers (Figure 4A). Expression of *rudhira*-RNAi-a using pan-neuronal *elav*-Gal4 caused lethality at the embryonic stage at 25°C and 29°C, and expression of *rudhira*-RNAi-b at 25°C caused lethality at the larval/pupal stage. Using a second pan-neuronal driver, *Appl*-Gal4,[30] it was possible to obtain adult flies at 25°C with both *rudhira*-RNAi lines, while at 29°C, expression of *rudhira*-RNAi-a led to paralyzed flies that died on the first day after eclosion. The difference between the two pan-neuronal drivers can possibly be explained by the onset and strength of driver expression. High-throughput RNA-seq expression patterns indicate that *Appl* is active at a later stage of development compared to *Elav*.[31] Knockdown of *rudhira* in motor neurons (*D42*-Gal4) at 25°C caused lethality at the larval (*rudhira*-RNAi-a line) or at the larval/pupal stage (*rudhira*-RNAi-b line), while lethality was shifted to the embryonic stage with the *rudhira*-RNAi-a line at 29°C. The

A

Driver	Expression	rudhira-RNAi-a, 25°C, lethality	rudhira-RNAi-a, 29°C, lethality	rudhira-RNAi-b, 25°C, lethality
control	n/a	-	-	-
elav	Pan-neuronal	Embryonic	Embryonic	Larval/Pupal
D42	Motor neuronal	Larval	Embryonic	Larval/Pupal
Appl	Pan-neuronal	-	Day after eclosion	-

B

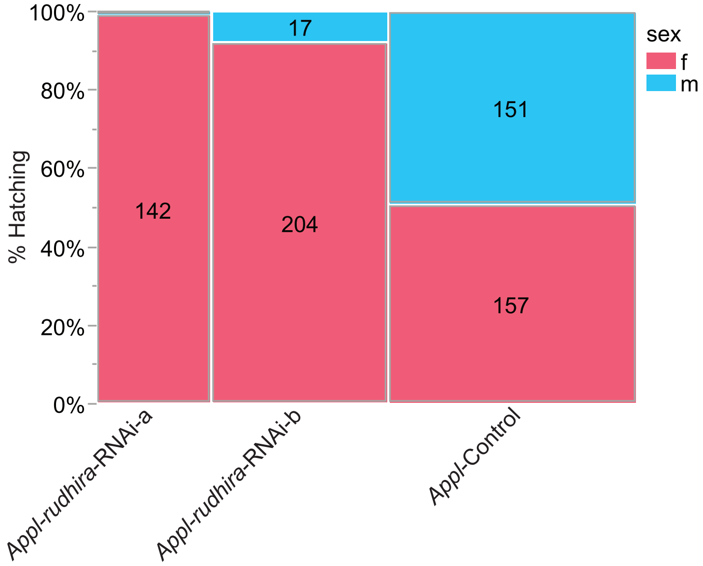

Figure 4. *Drosophila* loss-of-function model of *rudhira*

(A) Lethality screening of *rudhira* knockdown with *rudhira*-RNAi-a at 25°C and 29°C and *rudhira*-RNAi-b at 25°C under various Gal4 lines.

(B) Shifted female-to-male ratio of hatching flies when crossing *Appl*-Gal4 homozygous female virgin flies with UAS-*rudhira*-RNAi-a or UAS-*rudhira*-RNAi-b males (142 female and one male *Appl-rudhira*-RNAi-a flies, 204 female and 17 male *Appl-rudhira*-RNAi-b flies), while a control stock hatched at approximately equal ratios (157 female and 151 male *Appl*-control flies). χ^2 = 174.47, $p < 0.001$.

shift in lethality at higher temperatures to an earlier time point is most likely explained by the temperature-dependent efficiency of transgene expression of the UAS-Gal4 system.

Gene dosage compensation in *Drosophila* adjusts the expression of X chromosome genes by doubling expression in males.[32,33] Hence, the X chromosome neuronal driver *Appl*-Gal4 should be expressed at higher levels in hemizygous *Appl*-Gal4/Y males than in their heterozygous *Appl*-Gal4/+ female counterparts. By setting up crosses between *Appl*-Gal4 homozygous female virgins with UAS-*rudhira*-RNAi-a or UAS-*rudhira*-RNAi-b males, we hypothesized that there would be a higher female-to-male ratio in the knockdown group than in the control group. Indeed, at 25°C, male *Appl-rudhira*-RNAi-a (*Appl-Gal4*/Y;+/+;UAS-rudhira-RNAi-a/+) or male *Appl-rudhira*-RNAi-b flies were rarely observed, unlike in an *Appl*-control cross where males and females occurred at Mendelian ratios (Figure 4B). From a total of 402 offspring collected from the *Appl*-control cross (*Appl*-Gal4 homozygous female virgin flies crossed with TRiP background males), there were 156 females (51%) and 151 males (49%) in the F1 generation. In contrast, from a total of 143 offspring collected by crossing *Appl*-Gal4 homozygous female virgin flies with UAS-*rudhira*-RNAi-a males, 142 were females (99.3%) and 1 (0.7%) was male. Similarly, out of a total of 221 progeny with the rudhira-RNAi-b line, 204 *Appl-rudhira*-RNAi-b females (92.3%) and only 17 *Appl-rudhira*-RNAi-b males (7.7%) were observed.

Detailed phenotyping of adult *Appl-rudhira*-RNAi-a flies obtained at 25°C revealed reduced longevity, severe locomotion defects and a wing phenotype (Figures S3D–S3G), whereas consistent with the late onset of *Appl* expression, no significant defects in larval locomotion or neuromuscular junction morphology were identified in *Appl-rudhira*-RNAi-a larvae (Figure S4). However, these findings need to be replicated in a second UAS-rudhira-RNAi line. Additional neuronal and nonneuronal Gal4 drivers caused lethality or tissue-specific phenotypes in the *rudhira*-RNAi-a line but were not tested in a second *rudhira*-RNAi line (Figure S3).

Taken together, experiments from the *rudhira*-RNAi-a line suggest that RNAi-mediated knockdown of *rudhira* causes developmental defects in neuronal and non-neuronal tissues in a dose-dependent manner. The lethality of pan-neuronal and motor-neuronal knockdown was confirmed with the *rudhira*-RNAi-b line.

Discussion

In this study, we provide evidence that bi-allelic variants of *BCAS3* cause a syndromic neurodevelopmental disorder. We have described 15 probands from eight unrelated families with ten different bi-allelic germline variants of *BCAS3*. The associated syndromic phenotype comprises GDD with severe ID, microcephaly, a short stature, a movement disorder with pyramidal tract involvement, strabismus, and seizures in many cases. Furthermore, similar mild facial dysmorphic features, including a long face, tooth abnormalities, full lips with an everted lower lip, and a short philtrum, were present in almost all examined probands. Neuroradiologically, in addition to different

degrees of myelination deficits in younger probands and mild to moderate global atrophy in older probands, all probands showed a dysgenic corpus callosum with isolated or pronounced involvement of the splenium. These syndromic features should help to identify and diagnose more probands with pathogenic or likely pathogenic bi-allelic *BCAS3* variants.

Of note, in addition to the homozygous *BCAS3* variant, family 7 was found to have a homozygous variant in *HELZ* (MIM: 606699, c.3322A>G [p.Ile1108Val] [GenBank: NM_014877]), which was published as a candidate DD/ID-associated gene as part of a large DD/ID cohort (identifier BAB4698).[12] Mutation prediction tools, including PolyPhen, SIFT, and LRT, predicted the *HELZ* variant as benign, neutral, and tolerated, respectively. Additionally, the CADD score of the *HELZ* variant was 15.45. Given the obvious phenotypic similarity between affected individuals of family 7 and other subjects with *BCAS3* variants in this manuscript and the low prediction scores of likelihood for damaging effects of the given *HELZ* variant (in contrast to the *BCAS3* variant, which results in a frameshift at codon 177), we consider the identified *BCAS3* variant as the major driver of the probands' phenotype.

Based on the bi-allelic nonsense variants identified in most probands, we suspected the disease mechanism to be loss of function. By showing that BCAS3 was absent in both immunoblot and nanoLC-MS/MS analyses in fibroblasts from proband F4-II.1 carrying bi-allelic missense variants, we strengthened this hypothesis and confirmed the missense alleles as likely damaging and probably disease causing. Data from the knockout mouse model[2] and similarities of the phenotypes between *CDC42* (MIM: 116952)- and *BCAS3*-associated disease suggested a close regulatory interplay between BCAS3 and CDC42. Heterozygous missense variants in *CDC42* have recently been associated with a heterogeneous developmental disorder that includes variable growth dysregulation, facial dysmorphism, and neurodevelopmental, immunological, and hematological abnormalities (MIM: 616737, Takenouchi-Kosaki syndrome).[34] Interestingly, RNA-seq and nanoLC-MS/MS data based on proband's fibroblasts showed expression levels of CDC42 as well as of several other previously proposed interaction partners mostly unchanged. Nevertheless, the broad categories of pathways resulting from transcriptomics and proteomics ORA were similar to previous transcriptomics analyses from the *Bcas3* knockout mouse model and "extracellular matrix organization" and "angiogenesis" were the most enriched and most significant hits. One major limitation of this analysis is the limited comparability of proband's fibroblasts to the previously analyzed mouse yolk sac. Notably, fibroblasts and the presumably most disease-relevant cell line from the mouse model, endothelial cells, are derived from primitive mesenchyme.

The pathophysiological consequences of loss-of-function mutations in *BCAS3* seem to be potentially different in mice and humans. Compared to phenotypes observed in the knockout mouse, diseases in humans resulting from a loss of BCAS3 were less severe. While knockout leads to disorganized vessels in the brain, yolk sac, cardiovascular malformation, and embryonic death in knockout mice, the probands reported herein were born at term with normal weight and without major maldevelopment. There is no clear evidence of cardiovascular malformations in humans. Only asymptomatic bicuspid aortic valves were detected by echocardiography in two probands. Interestingly, in one of the 13 examined MRIs, a vein of Galen malformation was identified. TOF-MRA images of three other probands revealed normally developed large blood vessels in the brain. In summary, cardiovascular or vascular malformations in the brain do not appear to be a relevant part of the human phenotype.

Similarly, cell migration does not seem to be equally affected in humans as in mice. Endothelial cell migration of *Bcas3* knockout cells was strongly impaired and has been proposed to be one key component that contributes to impaired angiogenesis. Therefore, we would have expected probands' fibroblast cell lines to show reduced cell migration in the migration assays. However, migration was not impaired in proband cells compared to in control cell lines, with the obvious limitation that human endothelial cell lines might behave differently. In summary, the phenotype in humans resulting from loss of *BCAS3* is less severe than that in mice, and angiogenesis does not seem to be a major component of human pathogenesis.

The *Drosophila* loss-of-function model showed lethality in two different RNAi lines using pan-neuronal and motor-neuronal drivers. The adult flies that hatched under the X chromosome *Appl* driver were mostly female, indicating a dose dependency. Similarly, shifted time points of lethality under a higher temperature hints to a dose-dependent effect, although this was only explored in one RNAi line. Additional results showing specific phenotypes for different neuronal and nonneuronal drivers such as locomotion defects and abnormal wing postures for the *Appl* driver or the rough eye phenotype (using the eye-specific *GMR* driver) were based on the *rudhira*-RNAi-a line but must be interpreted with some caution as no investigation of a second RNAi line has been performed to confirm these findings.

Our data suggest an essential role for BCAS3/Rudhira in the development of neuronal cell populations that appear to be independent of angiogenesis and cell migration. We encourage follow-up studies based on neuronal cell models to explore the developmental biology and the human pathomechanisms of this Mendelian disorder.

Data and code availability

The data that support the findings of this study are available from the corresponding authors upon request. Human variants and phenotypes have been deposited in the ClinVar database (https://www.ncbi.nlm.nih.gov/clinvar) under the submission name ClinVar: BCAS3_0001. The respective ClinVar accession numbers can be found in Table S1.

Supplemental information

Supplemental information can be found online at https://doi.org/10.1016/j.ajhg.2021.04.024.

Consortia

The members of the Care4Rare Canada Consortium are Kym M. Boycott, Michael Brudno, Francois P. Bernier, Clara D. van Karnebeek, David A. Dyment, Kristin D. Kernohan, Micheil A. Innes, Ryan E. Lamont, Jillian S. Parboosingh, Deborah A. Marshall, Christian R. Marshall, Roberto Mendoza-Londono, James J. Dowling, Robin Z. Hayeems, Bartha M. Knoppers, Anna M. Lehman, and Sara A. Mostafavi. The members of the Genomics England Research Consortium are John C. Ambrose, Prabhu Arumugam, Marta Bleda, Freya Boardman-Pretty, Christopher R. Boustred, Helen Brittain, Mark J. Caulfield, Georgia C. Chan, Greg Elgar, Tom Fowler, Adam Giess, Angela Hamblin, Shirley Henderson, Tim J.P. Hubbard, Rob Jackson, Louise J. Jones, Dalia Kasperaviciute, Melis Kayikci, Athanasios Kousathanas, Lea Lahnstein, Sarah E.A. Leigh, Ivonne U.S. Leong, Javier F. Lopez, Fiona Maleady-Crowe, Loukas Moutsianas, Michael Mueller, Nirupa Murugaesu, Anna C. Need, Peter O'Donovan, Chris A. Odhams, Christine Patch, Mariana Buongermino Pereira, Daniel Perez-Gil, John Pullinger, Tahrima Rahim, Augusto Rendon, Tim Rogers, Kevin Savage, Kushmita Sawant, Richard H. Scott, Afshan Siddiq, Alexander Sieghart, Samuel C. Smith, Alona Sosinsky, Alexander Stuckey, Mélanie Tanguy, Ellen R.A. Thomas, Simon R. Thompson, Arianna Tucci, Matthew J. Welland, Eleanor Williams, Katarzyna Witkowska, Suzanne M. Wood, and Magdalena Zarowiecki.

Acknowledgments

We are grateful to the affected individuals and their families whose cooperation made this study possible. The study has been supported by the Deutsche Forschungsgemeinschaft (DFG, grant SCHO754/2-1 to L.S.) and funding of the NGS Competence Center Tübingen (INST 37/1049-1), the Solve-RD project (grant 779257 to H. Hengel, A.J., and L.S.), the US National Human Genome Research Institute (NHGRI) and National Heart Lung and Blood Institute (NHLBI) to the Baylor-Hopkins Center for Mendelian Genomics (BHCMG, UM1 HG006542, J.R.L.), the US National Institute of Neurological Disorders and Stroke (NINDS) (R35NS105078 to J.R.L.), and the National Institute for Health Research (NIHR) Oxford Biomedical Research Centre Programme and made possible through access to the data and findings generated by the 100,000 Genomes Project (managed by Genomics England Limited and funded by the NIHR and NHS England), by the Care4Rare Canada Consortium funded by Genome Canada and the Ontario Genomics Institute (OGI-147), the Canadian Institutes of Health Research, Ontario Research Fund, Genome Alberta, Genome British Columbia, Genome Quebec, and Children's Hospital of Eastern Ontario Foundation, and by the NIH R01 (2R01NS058721-07A1). H. Hengel was supported by the fortüne program of the University of Tübingen (grant #2554-0-0). H. Hengel and L.S. are members of the European Reference Network for Rare Neurological Diseases (project ID 739510). D.P. was supported by a Clinical Research Training Scholarship in Neuromuscular Disease partnered by the American Brain Foundation and Muscle Study Group and International Rett Syndrome Foundation (IRSF grant #3701-1). T.B.H. was supported by the DFG (project number 418081722). The families were collected as part of the SYNaPS Study Group collaboration funded by the Wellcome Trust and strategic award (Synaptopathies) funding (WT093205 MA and WT104033AIA).

Declaration of interests

J.R.L. has stock ownership in 23andMe, is a paid consultant for Regeneron Genetics Center, and is a co-inventor on multiple United States and European patents related to molecular diagnostics for inherited neuropathies, eye diseases, and bacterial genomic fingerprinting. The Department of Molecular and Human Genetics at Baylor College of Medicine receives revenue from clinical genetic testing conducted at Baylor Genetics (BG) Laboratories; J.R.L. is a member of the Scientific Advisory Board of BG Laboratories. A.B. is an employee of GeneDx. All other authors declare no competing interests.

Received: December 1, 2020
Accepted: April 29, 2021
Published: May 21, 2021

Web resources

ClinVar, https://www.ncbi.nlm.nih.gov/clinvar/
GenBank, https://www.ncbi.nlm.nih.gov/genbank/
gnomAD server, https://gnomad.broadinstitute.org
OMIM, https://omim.org/
WebGestalt, http://www.webgestalt.org/

References

1. Siva, K., Venu, P., Mahadevan, A., S K, S., and Inamdar, M.S. (2007). Human BCAS3 expression in embryonic stem cells and vascular precursors suggests a role in human embryogenesis and tumor angiogenesis. PLoS ONE *2*, e1202.
2. Shetty, R., Joshi, D., Jain, M., Vasudevan, M., Paul, J.C., Bhat, G., Banerjee, P., Abe, T., Kiyonari, H., VijayRaghavan, K., and Inamdar, M.S. (2018). Rudhira/BCAS3 is essential for mouse development and cardiovascular patterning. Sci. Rep. *8*, 5632.
3. Bärlund, M., Monni, O., Kononen, J., Cornelison, R., Torhorst, J., Sauter, G., Kallioniemi, A.; and Kallioniemi OLLI-P (2000). Multiple genes at 17q23 undergo amplification and overexpression in breast cancer. Cancer Res. *60*, 5340–5344.
4. Nikpay, M., Goel, A., Won, H.H., Hall, L.M., Willenborg, C., Kanoni, S., Saleheen, D., Kyriakou, T., Nelson, C.P., Hopewell, J.C., et al. (2015). A comprehensive 1,000 Genomes-based genome-wide association meta-analysis of coronary artery disease. Nat. Genet. *47*, 1121–1130.
5. Li, C., Li, Z., Liu, S., Wang, C., Han, L., Cui, L., Zhou, J., Zou, H., Liu, Z., Chen, J., et al. (2015). Genome-wide association analysis identifies three new risk loci for gout arthritis in Han Chinese. Nat. Commun. *6*, 7041.
6. Hu, H., Kahrizi, K., Musante, L., Fattahi, Z., Herwig, R., Hosseini, M., Oppitz, C., Abedini, S.S., Suckow, V., Larti, F., et al. (2019). Genetics of intellectual disability in consanguineous families. Mol. Psychiatry *24*, 1027–1039.
7. Makrythanasis, P., Maroofian, R., Stray-Pedersen, A., Musaev, D., Zaki, M.S., Mahmoud, I.G., Selim, L., Elbadawy, A., Jhangiani, S.N., Coban Akdemir, Z.H., et al. (2018). Biallelic variants in KIF14 cause intellectual disability with microcephaly. Eur. J. Hum. Genet. *26*, 330–339.

8. Hengel, H., Buchert, R., Sturm, M., Haack, T.B., Schelling, Y., Mahajnah, M., Sharkia, R., Azem, A., Balousha, G., Ghanem, Z., et al. (2020). First-line exome sequencing in Palestinian and Israeli Arabs with neurological disorders is efficient and facilitates disease gene discovery. Eur. J. Hum. Genet. *28*, 1034–1043.
9. Hawer, H., Mendelsohn, B.A., Mayer, K., Kung, A., Malhotra, A., Tuupanen, S., Schleit, J., Brinkmann, U., and Schaffrath, R. (2020). Diphthamide-deficiency syndrome: a novel human developmental disorder and ribosomopathy. Eur. J. Hum. Genet. *28*, 1497–1508.
10. Kremer, L.S., Bader, D.M., Mertes, C., Kopajtich, R., Pichler, G., Iuso, A., Haack, T.B., Graf, E., Schwarzmayr, T., Terrile, C., et al. (2017). Genetic diagnosis of Mendelian disorders via RNA sequencing. Nat. Commun. *8*, 15824.
11. Monies, D., Abouelhoda, M., AlSayed, M., Alhassnan, Z., Alotaibi, M., Kayyali, H., Al-Owain, M., Shah, A., Rahbeeni, Z., Al-Muhaizea, M.A., et al. (2017). The landscape of genetic diseases in Saudi Arabia based on the first 1000 diagnostic panels and exomes. Hum. Genet. *136*, 921–939.
12. Karaca, E., Harel, T., Pehlivan, D., Jhangiani, S.N., Gambin, T., Coban Akdemir, Z., Gonzaga-Jauregui, C., Erdin, S., Bayram, Y., Campbell, I.M., et al. (2015). Genes that Affect Brain Structure and Function Identified by Rare Variant Analyses of Mendelian Neurologic Disease. Neuron *88*, 499–513.
13. Turnbull, C., Scott, R.H., Thomas, E., Jones, L., Murugaesu, N., Pretty, F.B., Halai, D., Baple, E., Craig, C., Hamblin, A., et al.; 100 000 Genomes Project (2018). The 100 000 Genomes Project: bringing whole genome sequencing to the NHS. BMJ *361*, k1687.
14. Chen, X., Schulz-Trieglaff, O., Shaw, R., Barnes, B., Schlesinger, F., Källberg, M., Cox, A.J., Kruglyak, S., and Saunders, C.T. (2016). Manta: rapid detection of structural variants and indels for germline and cancer sequencing applications. Bioinformatics *32*, 1220–1222.
15. Guillen Sacoto, M.J., Tchasovnikarova, I.A., Torti, E., Forster, C., Andrew, E.H., Anselm, I., Baranano, K.W., Briere, L.C., Cohen, J.S., Craigen, W.J., et al.; Undiagnosed Diseases Network (2020). De Novo Variants in the ATPase Module of MORC2 Cause a Neurodevelopmental Disorder with Growth Retardation and Variable Craniofacial Dysmorphism. Am. J. Hum. Genet. *107*, 352–363.
16. Borchert, N., Dieterich, C., Krug, K., Schütz, W., Jung, S., Nordheim, A., Sommer, R.J., and Macek, B. (2010). Proteogenomics of Pristionchus pacificus reveals distinct proteome structure of nematode models. Genome Res. *20*, 837–846.
17. Rappsilber, J., Mann, M., and Ishihama, Y. (2007). Protocol for micro-purification, enrichment, pre-fractionation and storage of peptides for proteomics using StageTips. Nat. Protoc. *2*, 1896–1906.
18. Kliza, K., Taumer, C., Pinzuti, I., Franz-Wachtel, M., Kunzelmann, S., Stieglitz, B., Macek, B., and Husnjak, K. (2017). Internally tagged ubiquitin: a tool to identify linear polyubiquitin-modified proteins by mass spectrometry. Nat. Methods *14*, 504–512.
19. Cox, J., and Mann, M. (2008). MaxQuant enables high peptide identification rates, individualized p.p.b.-range mass accuracies and proteome-wide protein quantification. Nat. Biotechnol. *26*, 1367–1372.
20. Cox, J., Neuhauser, N., Michalski, A., Scheltema, R.A., Olsen, J.V., and Mann, M. (2011). Andromeda: a peptide search engine integrated into the MaxQuant environment. J. Proteome Res. *10*, 1794–1805.
21. Elias, J.E., and Gygi, S.P. (2007). Target-decoy search strategy for increased confidence in large-scale protein identifications by mass spectrometry. Nat. Methods *4*, 207–214.
22. Schwanhäusser, B., Busse, D., Li, N., Dittmar, G., Schuchhardt, J., Wolf, J., Chen, W., and Selbach, M. (2011). Global quantification of mammalian gene expression control. Nature *473*, 337–342.
23. Luber, C.A., Cox, J., Lauterbach, H., Fancke, B., Selbach, M., Tschopp, J., Akira, S., Wiegand, M., Hochrein, H., O'Keeffe, M., and Mann, M. (2010). Quantitative proteomics reveals subset-specific viral recognition in dendritic cells. Immunity *32*, 279–289.
24. Liao, Y., Wang, J., Jaehnig, E.J., Shi, Z., and Zhang, B. (2019). WebGestalt 2019: gene set analysis toolkit with revamped UIs and APIs. Nucleic Acids Res. *47* (W1), W199–W205.
25. Karczewski, K.J., Francioli, L.C., Tiao, G., Cummings, B.B., Alföldi, J., Wang, Q., Collins, R.L., Laricchia, K.M., Ganna, A., Birnbaum, D.P., et al.; Genome Aggregation Database Consortium (2020). The mutational constraint spectrum quantified from variation in 141,456 humans. Nature *581*, 434–443.
26. Sobreira, N., Schiettecatte, F., Valle, D., and Hamosh, A. (2015). GeneMatcher: a matching tool for connecting investigators with an interest in the same gene. Hum. Mutat. *36*, 928–930.
27. Song, X., Beck, C.R., Du, R., Campbell, I.M., Coban-Akdemir, Z., Gu, S., Breman, A.M., Stankiewicz, P., Ira, G., Shaw, C.A., and Lupski, J.R. (2018). Predicting human genes susceptible to genomic instability associated with *Alu*/*Alu*-mediated rearrangements. Genome Res. *28*, 1228–1242.
28. Brand, A.H., and Perrimon, N. (1993). Targeted gene expression as a means of altering cell fates and generating dominant phenotypes. Development *118*, 401–415.
29. Duffy, J.B. (2002). GAL4 system in Drosophila: a fly geneticist's Swiss army knife. Genesis *34*, 1–15.
30. Torroja, L., Chu, H., Kotovsky, I., and White, K. (1999). Neuronal overexpression of APPL, the Drosophila homologue of the amyloid precursor protein (APP), disrupts axonal transport. Curr. Biol. *9*, 489–492.
31. Gelbart, W.M., and Emmert, D.B. (2013). FlyBase High Throughput Expression Pattern Data. (FlyBase).
32. Breen, T.R., and Lucchesi, J.C. (1986). Analysis of the dosage compensation of a specific transcript in Drosophila melanogaster. Genetics *112*, 483–491.
33. Cline, T.W., and Meyer, B.J. (1996). Vive la différence: males vs females in flies vs worms. Annu. Rev. Genet. *30*, 637–702.
34. Martinelli, S., Krumbach, O.H.F., Pantaleoni, F., Coppola, S., Amin, E., Pannone, L., Nouri, K., Farina, L., Dvorsky, R., Lepri, F., et al.; University of Washington Center for Mendelian Genomics (2018). Functional Dysregulation of CDC42 Causes Diverse Developmental Phenotypes. Am. J. Hum. Genet. *102*, 309–320.

The American Journal of Human Genetics, Volume *108*

Supplemental information

Bi-allelic loss-of-function variants in *BCAS3* cause a syndromic neurodevelopmental disorder

Holger Hengel, Shabab B. Hannan, Sarah Dyack, Sara B. MacKay, Ulrich Schatz, Martin Fleger, Andreas Kurringer, Ghassan Balousha, Zaid Ghanim, Fowzan S. Alkuraya, Hamad Alzaidan, Hessa S. Alsaif, Tadahiro Mitani, Sevcan Bozdogan, Davut Pehlivan, James R. Lupski, Joseph J. Gleeson, Mohammadreza Dehghani, Mohammad Y.V. Mehrjardi, Elliott H. Sherr, Kendall C. Parks, Emanuela Argilli, Amber Begtrup, Hamid Galehdari, Osama Balousha, Gholamreza Shariati, Neda Mazaheri, Reza A. Malamiri, Alistair T. Pagnamenta, Helen Kingston, Siddharth Banka, Adam Jackson, Mathew Osmond, Care4Rare Canada Consortium, Genomics England Research Consortium, Angelika Rieß, Tobias B. Haack, Thomas Nägele, Stefanie Schuster, Stefan Hauser, Jakob Admard, Nicolas Casadei, Ana Velic, Boris Macek, Stephan Ossowski, Henry Houlden, Reza Maroofian, and Ludger Schöls

Figure S1

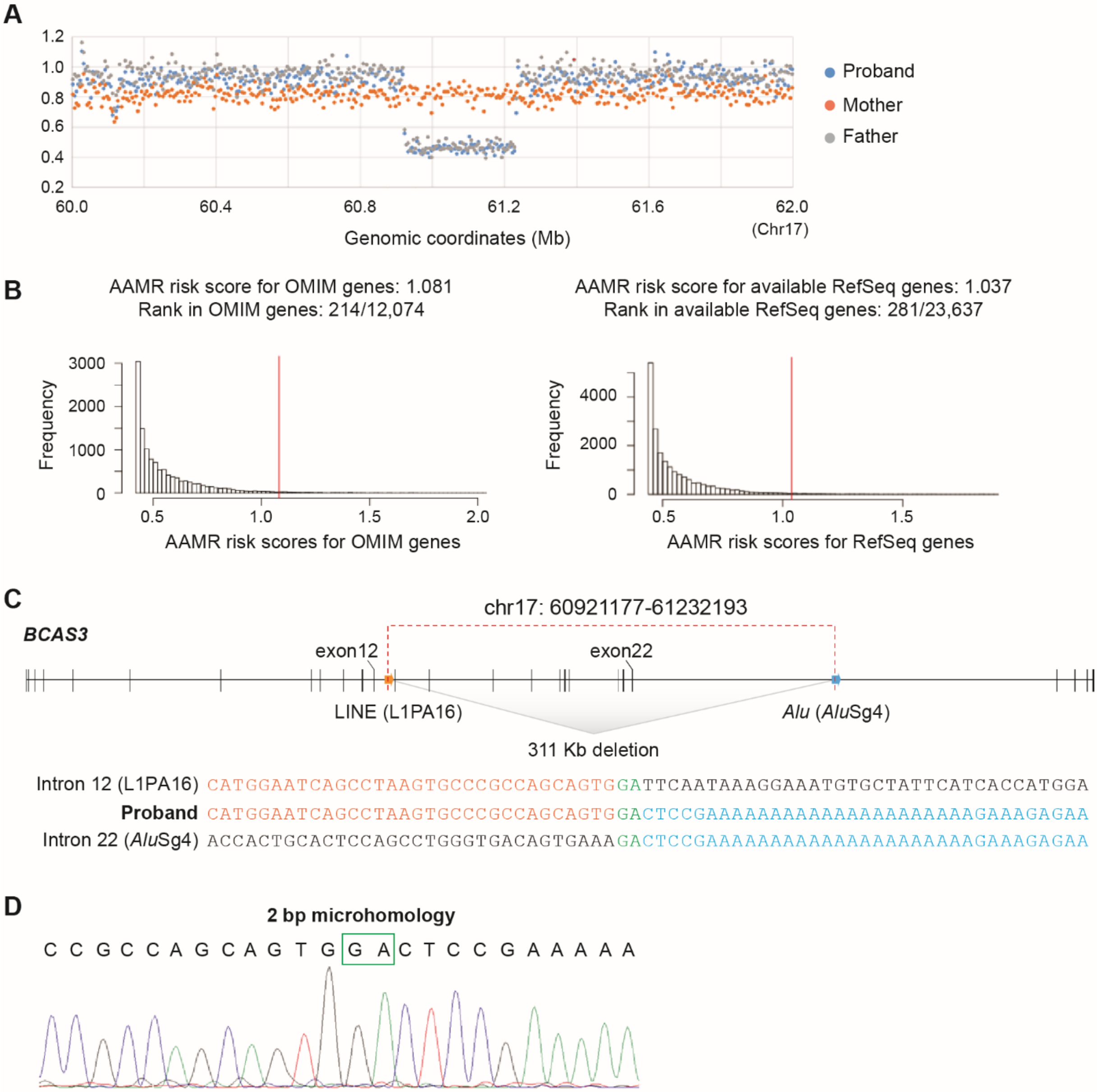

A
1.2
1.0
0.8
0.6
0.4
0.2
60.0
60.4
60.8
61.2
61.6
62.0
(Chr17)
Genomic coordinates (Mb)
Proband
Mother
Father
B
AAMR risk score for OMIM genes: 1.081
Rank in OMIM genes: 214/12,074
AAMR risk score for available RefSeq genes: 1.037
Rank in available RefSeq genes: 281/23,637
Frequency
3000
2000
1000
0
0.5
1.0
1.5
2.0
AAMR risk scores for OMIM genes
Frequency
4000
2000
0
0.5
1.0
1.5
AAMR risk scores for RefSeq genes
C
chr17: 60921177-61232193
BCAS3
exon12
exon22
LINE (L1PA16)
Alu (AluSg4)
311 Kb deletion
Intron 12 (L1PA16) CATGGAATCAGCCTAAGTGCCCGCCAGCAGTGGATTCAATAAAGGAAATGTGCTATTCATCACCATGGA
Proband CATGGAATCAGCCTAAGTGCCCGCCAGCAGTGGACTCCGAAAAAAAAAAAAAAAAAAAAAGAAAGAGAA
Intron 22 (AluSg4) ACCACTGCACTCCAGCCTGGGTGACAGTGAAAGACTCCGAAAAAAAAAAAAAAAAAAAAAGAAAGAGAA
D
2 bp microhomology
C C G C C A G C A G T G G A C T C C G A A A A A

Figure S1: A 311 Kb exonic deletion CNV allele generated by *Alu* – LINE rearrangement. **a** The 311 kb deletion identified by whole genome sequencing in F8. The read counts for non-overlapping 5 kb windows across the region was normalized to a control subject and shows evidence for copy number loss across ~ 300 Kb genomic interval. **b** The AluAluCNVPredictor tool (http://alualucnvpredictor.research.bcm.edu:3838/)[1] revealed the *Alu-Alu* mediated rearrangement (AAMR) score, a potential measure of susceptibility to genomic instability based on repetitive sequence gene architecture for that given gene, for *BCAS3* to be 1.081 for OMIM genes and 1.037 for RefSeq genes; a score > 0.6 implicates susceptibility to AAMR. **c** Schematic representation of exonic structure of *BCAS3*. Breakpoint sequence analysis for *BCAS3* deletions using the MultAlin alignment tool[2] is also shown. Proximal reference sequence and patient breakpoint sequences that match the proximal reference sequence are shown in orange, the distal reference sequence and patient breakpoint sequences that match the distal reference sequence are shown in blue. Note, 2 bp microhomology (GA) at the junction (shown in green). The 311 Kb deletion involving 10 exons identified in F8-II.1 presumably results from a Microhomology-Mediated Break-Induced Replication (MMBIR) event[3] generating the *BCAS3* intragenic deletion. **d** Sanger confirmation of the break point junction in F8-II.1.

Figure S2

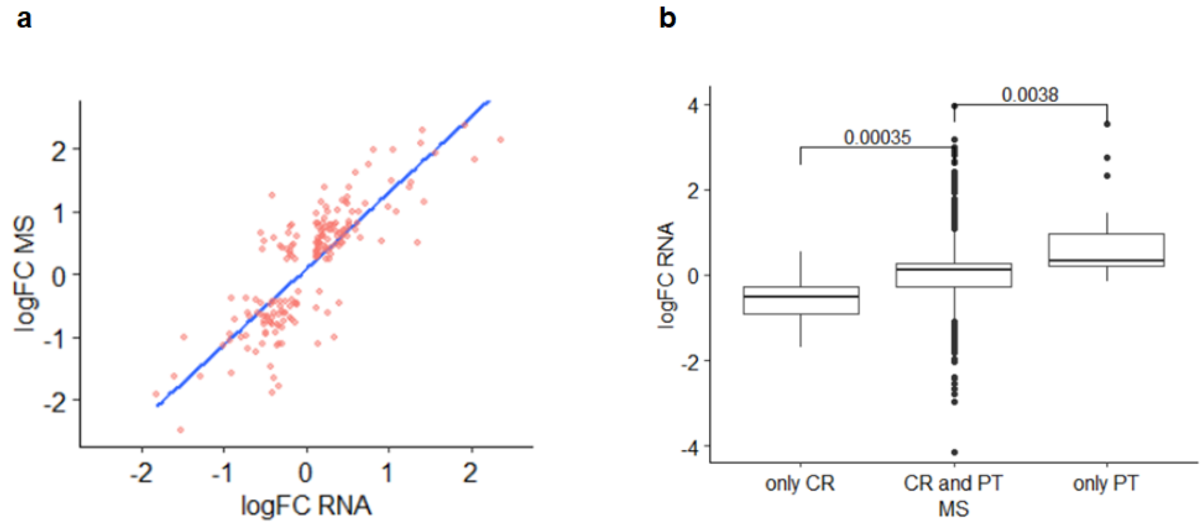

Figure S2: Correlation of RNA-Seq and NanoLC-MS/MS data

a Correlation of significant up/or downregulated genes at the mRNA level with significant up-/or downregulated proteins in MS. **b** Bar chart of fold changes in RNA-Seq data from proteins that were measured using nanoLC-MS/MS in only controls, in controls and patients and in only patients.

Figure S3

a

Driver	Expression(25°C) *rudhira* -RNAi-a	Lethality	Adult phenotype
control	n/a	-	Healthy
daughterless	Ubiquitous	Embryonic	-
elav	Pan-neuronal	Embryonic	-
Appl	Pan-neuronal	-	Weak, short-lived
GMR	Eye	-	Depigmentation
D42	Motor neuronal	Larval	-
ppk	Sensory neuronal	-	Healthy
repo	Pan-glial	Larval	-
Mhc	Muscles	Larval/Pupal	-

b

Driver	Expression (29°C) *rudhira-RNAi-a*	Lethality	Adult phenotype
control	n/a	-	Healthy
daughterless	Ubiquitous	Embryonic	-
elav	Pan-neuronal	Embryonic	-
Appl	Pan-neuronal	-	Paralyzed
GMR	Eye	Pupal	-
D42	Motor neuronal	Larval	-
ppk	Sensory neuronal	-	Healthy
repo	Pan-glial	Larval	-
Mhc	Muscles	Larval	-

c

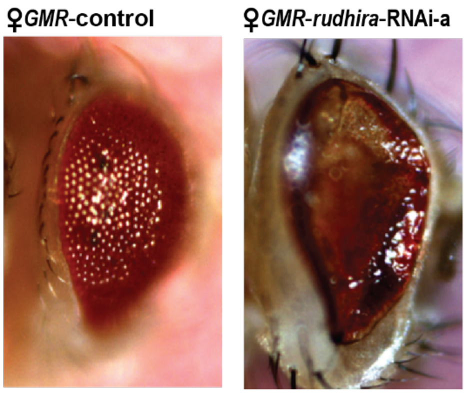

d

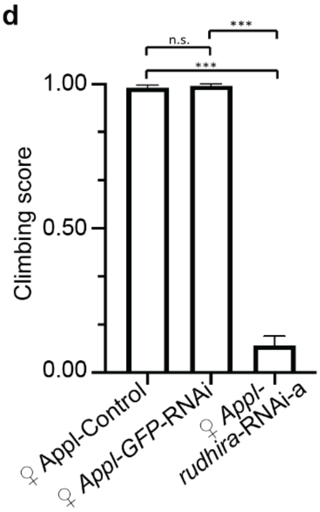

e

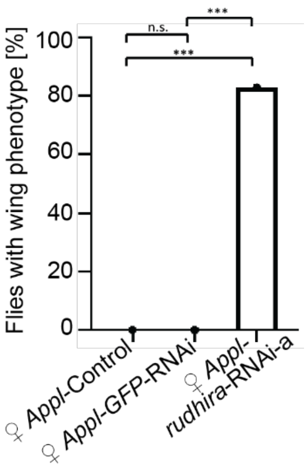

f

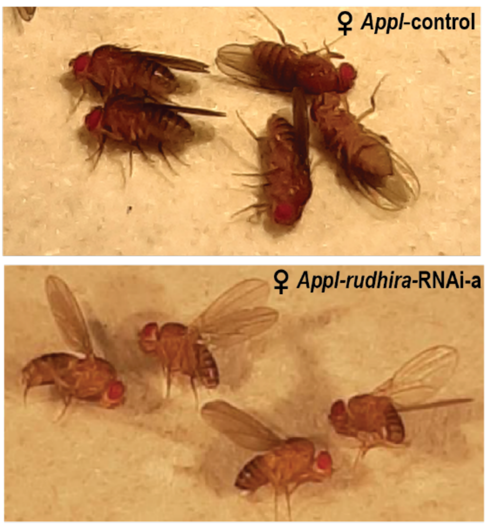

g

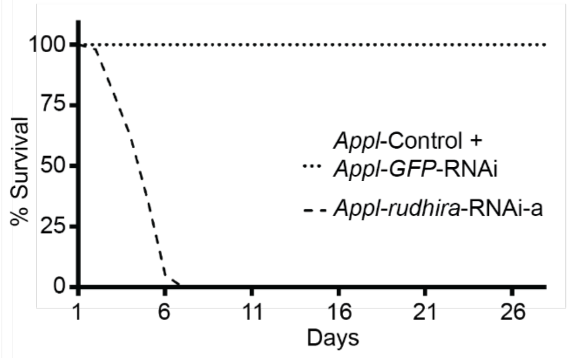

Figure S3: *Drosophila* loss-of-function model *based on the ruhdira-RNAi-a line.*

a Using the UAS-*rudhira*-RNAi-a line*,* knockdown of *rudhira* using different tissue specific neuronal and nonneuronal Gal4 driver lines at 25°C. Ubiquitous (*daughterless*), pan-neuronal (elav), motor neuroal (*D42*-Gal4), glial (*repo*-Gal4) and muscle (*Mhc*-Gal4) specific drivers caused lethality, whereas no effect was observed using a sensory neuron driver (*ppk*-Gal4). Adult flies with specific phenotypes were observed under the *Appl*-Gal4 (pan neuronal, active beginning in the larval stage) and *GMR*-Gal4 (eye-specific) drivers. **b** A higher temperature at 29°C shifted lethality to an earlier developmental stage. **c** Representative images of eyes when the Canton S line or *rudhira*-RNAi-a was crossed with the *GMR*-Gal4 driver at 25°C. The rough eye phenotype that was observed in *GMR-rudhira*-RNAi-a flies has been widely used to study the effect of gene knockdown or overexpression on neuronal dysfunction and neurodegeneration in *Drosophila*[4]. **d** Using a negative geotaxis assay, adult *Appl-rudhira*-RNAi-a and *Appl*-control flies were assayed for their ability to climb. *Appl-rudhira*-RNAi-a flies had a severe locomotion defect with impaired climbing abilities (Video S1-S3). Crossings were set up at 25°C. More than 50 flies were analyzed per genotype. **e** Impaired wing posture with wings perpendicular to the body in *Appl-ruhdira*-RNAi-a flies, while wings remained normal in *Appl*-control and *Appl*-GFP-RNAi flies. 120 *Appl*-control flies, 101 *Appl*-GFP-RNAi, and 128 *Appl-ruhdira*-RNAi-a were analyzed. **f** Representative images of the wing phenotype of *Appl-rudhira*-RNAi-a flies compared to *Appl*-control flies. **g** Compared to *Appl*-control and *Appl-GFP*-RNAi flies*, Appl-rudhira*-RNAi-a flies obtained at 25°C showed a reduced life span with a maximum life expectancy of 7 days. 100 flies were followed up in all three groups. While all *Appl-rudhira*-RNAI-a flies were dead after 7 days, all *Appl*-control or *Appl-GFP*-RNAi flies were still alive after 26 days of follow-up (log-rank $p<0.001$).

Figure S4

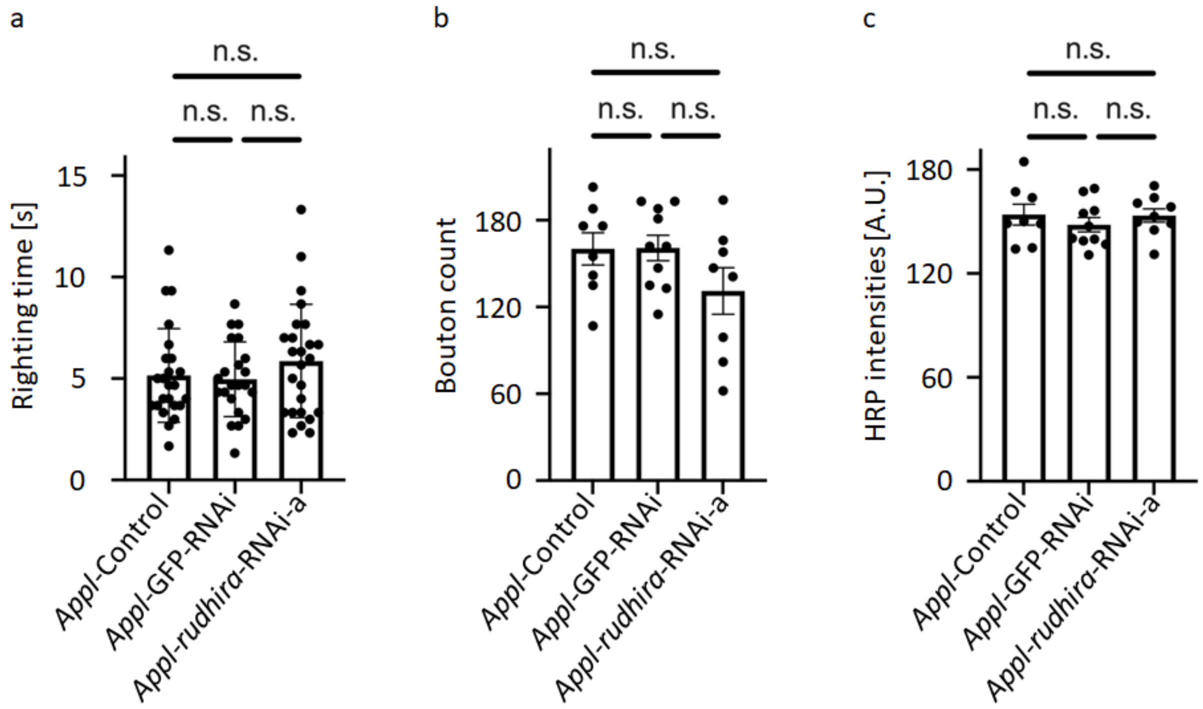

Figure S4: Characterization of control, GFP-RNAi, and *rudhira*-RNAi-a knockdown larvae using the *Appl*-Gal4 driver. **a** Comparison of the righting times between the *Appl*-control strain, *Appl*-GFP-RNAi and *Appl*-*rudhira*-RNAi-a larvae. The *Appl*-control group represents F1 female larvae following the cross between *Appl*-Gal4 with background control strain (BDSC #36304). *Appl*-*GFP*-RNAi group represents F1 female larvae following the cross between *Appl*-Gal4 with *GFP*-RNAi (BDSC #9331). More than 20 individual larvae were analyzed per genotype. No significant differences were observed between any groups with Kruskal-Wallis (p=0.62) followed by Dunn's multiple comparisons test, while there was a trend towards a slower righting time in the *Appl*-*rudhira*-RNAi-a larvae. **b** Bouton counts from *Appl*-control, *Appl*-*GFP*-RNAi and *Appl*-*rudhira*-RNAi-a larvae. N between 8-10 NMJs, n represents NMJs from distinct larvae. No significant differences were observed between any groups with Kruskal-Wallis (p=0.37) followed by Dunn's multiple comparisons test, while there was a trend towards less boutons in the *Appl*-*rudhira*-RNAi-a larvae. **c** HRP intensities quantified from neuromuscular junctions of *Appl*-control, *Appl*-*GFP*-RNAi and *Appl*-*rudhira*-RNAi-a larvae. N between 8-10 NMJs, n represents NMJs, each from a distinct larvae. No significant differences were observed between any groups with Kruskal-Wallis (p=0.61) followed by Dunn's multiple comparisons test **.**

Table S1: Genetic characteristics of identified BCAS3 variants

Family	Chr	Start	End	Ref	Alt	Variant (NM_001099432.3)	gnomAD (v2.1.1)	SIFT	Polyphen2 (HumVar)	CADD (phred)	GERP++	phyloP (100way)	ClinVar Accession
F1	17	60679530	60679530	C	T	c.73C>T, p.Gln25*	.	0	.	38	4.53	5.688	SCV001470694
F2	17	60889759	60889759	T	G	c.726T>G, p.Tyr242*	.	1	.	38	5.99	1.95	SCV001470695
F3	17	60990206	60990206	C	G	c.1457C>G, p.Ser486*	.	0.08	.	39	5.38	6.995	SCV001470696
F4	17	61034683	61034683	C	T	c.1700C>T, p.Pro567Leu	2.03E-05	0.3	0.997	26.4	6.06	7.487	SCV001470697
F4	17	61034712	61034712	G	A	c.1729G>A, p.Gly577Arg	1.61E-05	0.14	0.999	24.1	6.06	9.476	SCV001470698
F5	17	61078384	61078384	C	T	c.2227C>T, p.Gln743*	.	0.14	.	40	5.61	7.598	SCV001470699
F6	17	60947264	60947264	T	-	c.1133delT, p.Val378fs*5	.	.	.	.	.	.	SCV001470700
F7	17	60868629	60868629	T	-	c.530delT, p.Met177fs*23	.	.	.	.	.	.	SCV001470701
F8	17	60921177	61232193	311 kb deletion		c.994-3230_2426-136134del, p.?	.	.	.	.	.	.	SCV001470702
F8	17	61040896	61040899	AGTA	-	c.2074+4_2074+7delAGTA, p.?	.	.	.	.	.	.	SCV001470703
F9	17	60747213	60747213	C	T	c.337C>T, p.Gln113*	.	0.29	.	37	5.36	5.456	SCV001548239
F9	17	60868675	60868675	C	A	c.576C>A, p.Cys192*	.	1	.	37	2.59	2.724	SCV001548240
Candidate variant from Hu et al.	17	61084564	61084564	G	C	c.2470G>C, p.Gly824Arg	.	0.01	0.804	18.37	5.92	9.187	n/a

Table S2: Analysis and pathological findings in available MRI studies

Proband	F1-II.1	F1-II.2	F2-II.3	F2-II.2	F2-II.7	F3-II.1	F4-II.1	F5-II.1	F5-II.2	F6-II.2	F6-II.1	F6-II.1	F7-II.1	F7-II.2	F9-II.1
Age	9 yrs.	5 yrs.	21 yrs.	22 yrs.	7 yrs.	14 mo.	5 mo.	9 yrs.	4/1/2 yrs.	4 yrs.	1 yr.	7 yrs.	7 yrs.	6 yrs.	11 yrs.
Medulla oblongata	0	0	0	0	0	0	1	0	0	0	0	0	0	0	0
Pons	0	0	0	0	0	0	2	0	0	0	0	0	0	0	0
Mesencephalon	0	0	0	0	0	0	0	0	0	0	0	0	0	0	0
Cerebellar vermis	0	1	1	1	1	0	0	1	1	2	1	1	1	1	1
Cerbellar hemispheres	0	0	1	1	0	0	1	1	1	0	0	0	0	0	0
Corpus callosum genu	0	0	0	0	0	0	2	0	1	1	1	1	0	0	2
Corpus callosum body	1	1	1	1	1	0	2	0	2	1	1	1	1	0	2
Corpus callosum splenium	2	2	2	2	1	2	2	2	2	2	2	2	1	1	2
Frontal cortex	0	0	1	2	1	1	1	0	1	2	1	2	1	0	0
Parietal cortex	0	0	2	2	1	1	1	0	1	2	1	1	1	0	0
Occipital cortex	0	0	2	1	1	0	1	0	1	2	0	1	1	0	0
Temporal cortex	0	0	1	0	0	0	1	0	1	2	0	2	1	0	0
Myelination frontal	1	1	0	0	1	1	2	1	1	2	1	0	1	1	1
Myelination parietal	2	2	0	0	1	1	2	1	1	2	1	0	1	1	1
Myelination occipital	1	1	0	0	0	0	2	1	1	2	1	0	1	0	1
Myelination temporal	1	1	0	0	0	1	2	1	1	1	1	0	1	0	1
Basal ganglia	0	0	0	0	0	0	1	0	0	0	0	0	0	0	0
Angiography	n/a	n/a	0	0	0	n/a	n/a	n/a	Vein of Galen aneurysmal malformation	n/a	n/a	n/a	n/a	n/a	n/a

Evaluation of the pathological findings (atrophy or myelination deficits): 0 = normal, 1 = slightly pathological, 2 = clearly pathological.

Supplemental Methods

Drosophila husbandry and strains

The following additional Gal4 driver lines were used: *ppk*-Gal4 (BDSC #32078, #32079), *Mhc*-Gal4 (BDSC #55133), *GMR*-Gal4 (from Aaron Voigt, Department of Neurology in RWTH Aachen University) and *repo*-Gal4 (from Christian Klämbt, University of Münster).

Immunohistochemistry

Immunohistochemistry at the neuromuscular junction in aged-matched L3 larvae was performed as previously described [5, 6]. Only female larvae were used for dissection. In brief, larvae were removed from food with a brush and washed gently with tap water at room temperature. Larvae were then immobilized on a dissection pad with dissection pins and Ca2+ free HL-3 solution (on ice) were added to cover the larvae. Dissection scissors were used for dissection to expose body wall muscles by cutting along the dorsal side midline of the larvae. Next, inner organs were gently removed, HL-3 was removed and 4% paraformaldehyde in phosphate-buffered saline (PBS) was added for 10min for fixation. For blocking solution, PBS supplemented with 0.05% Triton X100 (PBT) was utilized, and 5% normalized goat serum in PBT was used (1 hour at room temperature). Goat antihorseradish peroxidase (HRP) conjugated with Cy3 (Dianova) was added and incubation performed for 2h at room temperature. HRP antibody incubation was performed was performed in 5% normalized goat serum in PBT. Samples were washed in PBS (3 times, 20 mins) prior to mounting in Vectashield (Vector Laboratories).

Microscopy and image analysis

Microscopy and image analysis were performed as previously described [7]. Fixed samples were imaged on a Zeiss LSM 710 confocal microscope equipped with 405, 440, 488, 514, 561 and 633 Laser Lines and ConfoCor 3 Scanhead. Imaging was performed using a 40X oil objective with 1.3 N. A.; Voxel Size: 100nm, 100nm, 500 nm; pinhole: 1 AU, average: 2. Samples were imaged while avoiding oversaturation of signals. For quantitative comparisons of intensities,

common settings were chosen for all parameters, including the speed of scanning, frame size, laser intensities, and digital gain. NMJ from muscle 6/7 in segment 3 from either the left or right hemisegment were imaged. Image processing at the larval neuromuscular junction was performed using the open source image processing software Fiji (https://imagej.net/Fiji, version 1.0). Boutons were counted manually using the HRP signal that stains neuronal membranes in nerve terminals. A binary 0-255 mask was generated using the HRP signal to quantify intensities. The same settings were used across all genotypes for generation of the HRP masks. Binary masks were used to do a minimum image calculation with HRP raw data that were background subtracted (the same background was subtracted across all genotypes). Intensities were analyzed from larvae that were dissected on the same day, incubated together in the same concentration of antibodies, and imaged using identical settings on the same imaging session at the confocal microscope. 8-10 NMJs from muscle 6/7 in segment 3, each from a distinct larvae, were analyzed per genotype.

Adult eye phenotypes

Three- to five-day-old adult flies from crosses between GMR-Gal4 with stock 51691 or Canton S as a control were frozen for 1 h at −20°C and then mounted on microscope slides. Eye pictures were taken using a Zeiss AxioImager Z1 microscope and AxioVision software.

Climbing assay

The climbing assay was performed as previously described[6, 8]. Flies were collected from all genotypes within 0-48h after eclosion. Flies were monitored by analyzing their ability to climb 6 cm in 14 s. . A successful attempt was scored as 1, and failure to reach the top as 0. Each fly was assessed three times to calculate the average climbing score (1 if all attempts three are successful, 0.67 if two out of three attempts are successful, and 0.33 if one out of three attempts are successful). Per genotype 50 female flies were analyzed.

Righting assay

Larval righting assay was performed as previously described[8, 9]. Only female L3 larvae from each genotype were used for the righting assay. In brief, larvae were removed from food and gently washed with tap water at room temperature. Before proceeding with the experiments, larvae were placed for 10min on an agar plate to acclimatize to experimental conditions. For the assay, larvae were turned one at a time and put upside down on agar plates. The time required to regain normal posture (righting) and initiate the first contraction wave was recorded using a stopwatch. Each larva was assayed three times, and the average righting time per trial was used for analysis. At least 20 larvae were analyzed per genotype.

Longevity assay

Emerged flies were collected within 24 h after eclosion and were kept at 25°C in a 12 h day/night cycle. Flies were transferred to vials containing fresh food media every day without anaesthetization. 100 flies were followed per genotype.

Supplemental References

1. Song X, Beck CR, Du R, et al. Predicting human genes susceptible to genomic instability associated with Alu/Alu-mediated rearrangements. Genome Res 2018;28(8):1228-1242.
2. Corpet F. Multiple sequence alignment with hierarchical clustering. Nucleic Acids Res 1988;16(22):10881-10890.
3. Mayle R, Campbell IM, Beck CR, et al. DNA REPAIR. Mus81 and converging forks limit the mutagenicity of replication fork breakage. Science 2015;349(6249):742-747.
4. Lenz S, Karsten P, Schulz JB, Voigt A. Drosophila as a screening tool to study human neurodegenerative diseases. J Neurochem 2013;127(4):453-460.
5. Zhang YV, Hannan SB, Kern JV, et al. The KIF1A homolog Unc-104 is important for spontaneous release, postsynaptic density maturation and perisynaptic scaffold organization. Sci Rep 2017;7:38172.
6. Zhu JY, Hannan SB, Drager NM, et al. Autophagy inhibition rescues structural and functional defects caused by the loss of mitochondrial chaperone Hsc70-5 in Drosophila. Autophagy 2021:1-15.
7. Kern JV, Zhang YV, Kramer S, Brenman JE, Rasse TM. The kinesin-3, unc-104 regulates dendrite morphogenesis and synaptic development in Drosophila. Genetics 2013;195(1):59-72.
8. Zhu JY, Vereshchagina N, Sreekumar V, et al. Knockdown of Hsc70-5/mortalin induces loss of synaptic mitochondria in a Drosophila Parkinson's disease model. PLoS One 2013;8(12):e83714.
9. Butzlaff M, Hannan SB, Karsten P, et al. Impaired retrograde transport by the Dynein/Dynactin complex contributes to Tau-induced toxicity. Human molecular genetics 2015;24(13):3623-3637.
